# Antibiotic nanozyme hydrogel depot for single-injection MDR bacterial keratitis therapy *via* localized antibacterial, pro-healing and corneal reinforcement

**DOI:** 10.1016/j.mtbio.2025.102602

**Published:** 2025-12-03

**Authors:** Hongwei Wang, Yuxin Liu, Li Ma, Xiaoyan Sun, Na Li, Xin Sui, Xia Qi, Shengqian Dou, Tan Li, Weiyun Shi, Ting Wang

**Affiliations:** a State Key Laboratory Cultivation Base, Shandong Key Laboratory of Eye Diseases, Eye Institute of Shandong First Medical University, Qingdao, China; b Eye Hospital of Shandong First Medical University, Jinan, China; c Qingdao Eye Hospital of Shandong First Medical University, Qingdao, China; d School of Ophthalmology, Shandong First Medical University, Jinan, China; e The First School of Clinical Medicine, Binzhou Medical University, Binzhou, China

**Keywords:** Hydrogel, Corneal cross-linking, Nanozyme, Multidrug-resistant, Bacterial keratitis

## Abstract

Bacterial keratitis (BK), a severe ocular infection caused by pathogenic bacterial invasion, requires urgent therapeutic development due to the limitations of conventional antibiotics, such as drug resistance and systemic toxicity caused by frequent dosing. To address these challenges, we developed an injectable antibiotic nanozyme hydrogel depot (ANHD) for single-dose therapy of BK. The ANHD was prepared by synthesizing the Ce-gatifloxacin nanozyme (CGN, ∼1.26 nm) through metal-organic coordination between cerium nitrate and gatifloxacin, followed by co-precipitation, and subsequently incorporating CGN into an injectable xanthan-PEG hydrogel constructed *via* Schiff base linkages. The resulting hierarchical composite exhibited multiple functional synergistic effects, including antibacterial activity, reactive oxygen species (O_2_^·-^/H_2_O_2_/·OH) scavenging activity, promotion of corneal wound healing, and reinforcement of corneal mechanical strength. Furthermore, nanozymes and hydrogels were ubiquitous in the corneal stroma and could exert their functions without penetration across the corneal surface, which distinguished them from the eye drop formulations. In a murine model, single injection of ANHD into corneal stroma effectively treated multidrug-resistant *Staphylococcus epidermidis* keratitis, with complete bacterial eradication, significant inflammation reduction, and accelerated epithelial repair. These results demonstrated the potential of ANHD in the treatment of ocular infectious diseases.

## Graphical abstract

**Figure:**
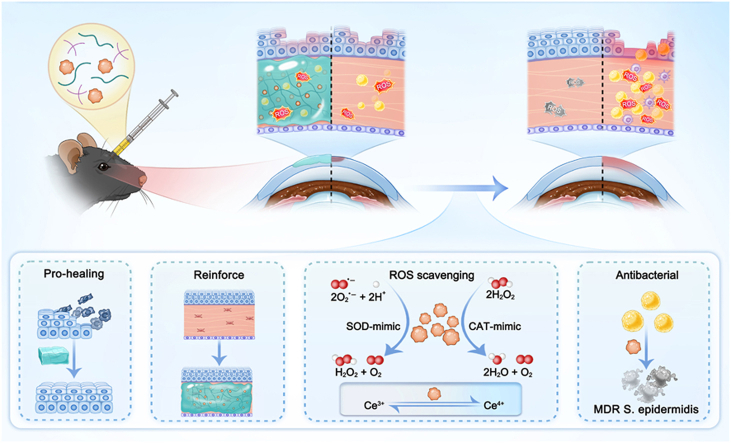

## 1 Introduction

Bacterial keratitis (BK) represents a severe ocular infection primarily caused by the invasion of pathogens such as Staphylococcus, Streptococcus, Pseudomonas, and Serratia species [1,2]. When corneal trauma or ocular pathologies compromise the corneal natural defense barriers, bacterial pathogens rapidly colonize the corneal tissue. These invading microorganisms produce an array of virulence factors, including toxins, proteases, and adhesins, that collectively drive the progression of aggressive infectious keratitis. The infectious process triggers a robust inflammatory response characterized by elevated reactive oxygen species (ROS) levels, leading to corneal tissue damage. Ocular redness, pain, and blurred vision are the typical clinical manifestations. Without prompt intervention, the disease may progress to severe complications such as corneal ulceration, scarring, perforation, and ultimately permanent vision loss [3,4]. Currently, fluoroquinolone antibiotics, including ofloxacin, levofloxacin, and ciprofloxacin, are FDA-approved commercial drugs for BK. However, the emergence of multidrug-resistant (MDR) strains leads to the limited efficacy of antibiotics against bacterial infections. In addition, the frequent dosing for local treatment often results in poor patient compliance, which highlights the urgent need to develop novel therapeutic strategies to combat the vision-threatening corneal infection [[5], [6], [7], [8], [9]].

Nanozymes, a class of nanomaterials mimicking natural enzyme catalytic activities, have emerged as promising candidates in biomedical applications due to their unique properties and versatile functionalities [[10], [11], [12], [13], [14], [15]]. These artificial enzymes primarily include metal-based, carbon-based, MOF-based, and single-atom nanozymes, which exhibit tunable catalytic activity, exceptional stability, and cost-effectiveness. Building upon these properties, we developed an active pharmaceutical ingredients-based nanozyme by integrating iron ions, proanthocyanidins, and the non-steroidal anti-inflammatory drug diclofenac sodium [16]. The designed nanozyme exhibits excellent biocompatibility, self-regulated redox capability, and remarkable anti-inflammatory activity. In corneal alkali burn models, the nanozyme demonstrated multifaceted therapeutic effects toward corneal neovascularization through efficient scavenging of ROS, suppression of inflammatory factors, promotion of angiogenic factors, and inhibition of ferroptosis, suggesting the promise of conventional antibiotic drugs as building blocks for developing nanozymes in ocular antibacterial applications. For instance, cerium nitrate and appropriate antibiotics are expected to produce dual-functional nanozymes with both antioxidant and antibacterial activities.

Hydrogels are a class of three-dimensional hydrophilic polymer networks [[17], [18], [19], [20]]. With exceptional biocompatibility, tunable physicochemical properties, and biodegradability, hydrogels are established as versatile platforms for diverse biomedical applications, including drug delivery systems, tissue engineering scaffolds, and wound dressings. In addition, they can serve as effective dispersion media for functional nanomaterials, ensuring the uniform distribution and avoiding the possible aggregation [[21], [22], [23], [24]]. When administered as eye drops, hydrogel formulations demonstrate distinct advantages for ocular therapy by significantly prolonging drug residence time on the ocular surface and enhancing bioavailability. However, nanomaterials in conventional hydrogel eye drops still face limitations in penetration across the intact corneal barrier. Intrastromal injection of Schiff base hydrogels represents an alternative approach to address this challenge. The reversible Schiff base bonds facilitate complete hydrogel degradation and subsequent nanomaterial clearance from corneal tissues, supporting long-term biosafety [[25], [26], [27], [28]].

Xanthan gum, one of the most important microbial polysaccharides, has been widely used in diverse fields, including food, biomedicine, pharmaceuticals, and agriculture [[29], [30], [31]]. Structurally, xanthan gum is a natural polysaccharide composed of repeated units of D-glucose, D-mannose, and D-glucuronic acid, along with side chains. The xanthan gum backbone contains abundant polar functional groups, such as hydroxyl and carboxyl groups, with *cis*-1,2-diol hydroxyl groups being particularly reactive. These groups can be readily oxidized by sodium periodate (NaIO_4_) to form aldehyde groups, serving as a suitable cross-linker for fabricating hydrogels. In addition to hydrogels, some other carriers such as nanoparticles and microcapsules can also be prepared using xanthan gum as the precursor. These carriers can be used to encapsulate both hydrophobic and hydrophilic drugs to develop advanced ocular drug delivery systems [[32], [33], [34], [35], [36], [37], [38], [39], [40], [41], [42], [43], [44]]. Owing to their minimal irritation and toxicity to ocular tissues, xanthan gum-based formulations, prolonging drug retention on the ocular surface and enhancing bioavailability, are effective for treating anterior segment diseases such as dry eye syndrome and keratitis.

Corneal cross-linking (CXL), initially developed as a photochemical treatment for corneal ectatic disorders, has been used to strengthen corneal biomechanics through collagen cross-linking using riboflavin and ultraviolet A [[45], [46], [47], [48]]. CXL has been successfully adapted for BK treatment by generating microbicidal ROS and enhancing stromal resistance to enzymatic degradation [49]. As a non-antibiotic antimicrobial approach, however, it faces limitations including pain and infection risks from epithelial removal, limited efficacy against deep infections due to poor ultraviolet A penetration, stromal cell damage, and lack of antimicrobial pharmaceutical ingredients. These limitations have stimulated researchers to develop new CXL approaches. Schiff base crosslinked hydrogel systems may provide mechanical support and deliver antibacterial and antioxidant nanoparticles during keratitis treatment, thereby preserving therapeutic benefits of CXL while overcoming the drawbacks of traditional ultraviolet A-riboflavin cross-linking to establish a safer, more effective BK therapy method [[50], [51], [52], [53], [54]].

**Scheme 1:**
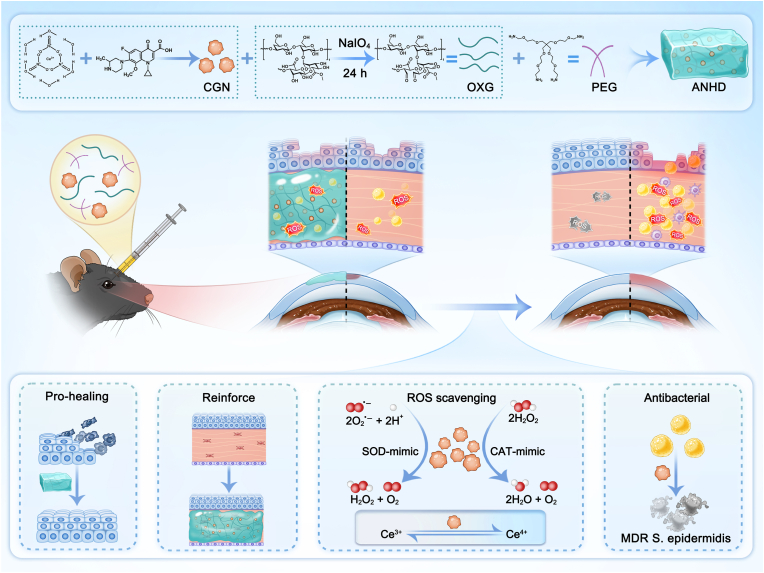
Schematic illustration of ANHD preparation and its application in BK therapy.

In the study, we proposed a single injection of antibiotic nanozyme hydrogel depot (ANHD) into the corneal stroma for *in situ* CXL to treat BK (Scheme 1). The hydrogel composite was prepared in two steps. Firstly, Ce-gatifloxacin nanozyme (CGN) was synthesized by metal-organic coordination of cerium nitrate and gatifloxacin at room temperature, followed by co-precipitation. Secondly, the xanthan-PEG hydrogel (XPH) was constructed through Schiff base condensation between oxidized xanthan gum (OXG) and 4-arm poly(ethylene glycol)-amine (4-arm PEG-NH_2_). The ANHD was subsequently prepared using the same method as XPH, except that CGN was incorporated into the hydrogel. The biomimetic enzymatic activity, drug release profile, and corneal mechanical strength, as well as its multifunctional therapeutic effects including antibacterial, antioxidant, and pro-healing capacities, were evaluated. Thereafter, we further investigated the efficacy of ANHD-mediated CXL approach in the treatment of MDR *S. epidermidis* keratitis.

## 2 Experimental section

### 2.1 Chemicals and reagents

Xanthan gum (acetylation degree, 2.9 %), cerium nitrate hexahydrate (99.5 %), gatifloxacin (98.0 %), ammonia (25 %–28 %), acetonitrile (99.5 %), acetic acid (99.5 %), ethylene glycol (98.0 %), NaIO_4_ (99.5 %), and 4-arm PEG-NH_2_ (98.0 %) were bought from Macklin (Shanghai, China). Hydroxyl radical assay kits and the inhibition and production of superoxide anion (O_2_^·-^) colorimetric assay kits were purchased from Nanjing Jiancheng Bioengineering Institute (Nanjing, China). Dihydroethidium (DHE) (98.0 %), hydrogen peroxide (H_2_O_2_) assay kit, and hydroxyl radical (·OH) assay kit were purchased from Solarbio (Beijing, China). Cell counting kit-8 (CCK-8) kits, live/dead (SYTO 9/PI) bacterial double stain kits, recombinant mouse tumor necrosis factor-α (TNF-α), interleukin-1β (IL-1β), and interleukin-6 (IL-6) enzyme-linked immunosorbent assay (ELISA) kits were manufactured by YaMei Biotechnology (Shanghai, China). DMEM/F12 culture medium, fetal bovine serum (FBS), and penicillin-streptomycin solution were purchased from Gibco (Gibco, USA). 0.25 % Trypsin-EDTA solution was bought from Sigma (Shanghai, USA).

### 2.2 Physical characterizations

The nanostructures of CGN were observed using transmission electron microscopy (TEM) with the NEC JEM-1200EX (Japan) instruments. Morphological characteristics of XPH and ANHD were observed using a GeminiSEM 360 scanning electron microscope (Zeiss, Germany). X-ray diffraction (XRD) analysis was carried out using a Rigaku SmartLab instrument (Japan). Elemental compositions of CGN, XPH, and ANHD were determined by X-ray photoelectron spectroscopy (XPS, ESCALAB-250Xi, USA). Rheological properties of XPH and ANHD were evaluated using an AR-G2 rheometer(USA). Zeta potential data were recorded using a Mastersizer 2000 laser diffractometer (Malvern Instruments, UK). Fourier-transform infrared spectroscopy (FT-IR) analysis was performed using a Bruker spectrometer (INVENIO S, Germany) with a wavelength range of 500–4000 cm^−1^. Optical transmittance was quantified across the wavelength range of 200–800 nm with a UV–Vis spectrophotometer (SpectraMax M2, Molecular Devices, USA). Molecular weight of xanthan gum was determined using a Waters 1515 GPC system (Waters, USA).

### 2.3 Material synthesis

#### 2.3.1 Synthesis of CGN

Cerium nitrate (291.0 mg) and gatifloxacin (250.0 mg) were dissolved in distilled water (25.0 mL), and the mixture was stirred at room temperature for 15 min. Subsequently, ammonia (3.0 mL) was added dropwise and the reaction was continued at room temperature for 12 h. Afterward, the mixture was centrifuged at 3000 rpm for 10 min. The collected supernatant was dialyzed (MWCO, 8–14 kDa) in distilled water for 2 days, and freeze-dried for 3 days to obtain the CGN.

#### 2.3.2 Fabrication of xanthan-PEG hydrogel (XPH)

Xanthan gum (3.0 g) and NaIO_4_ (1.8 g) were dissolved in 300.0 mL and 50.0 mL of distilled water, respectively. Two solutions were then transferred sequentially to a round-bottom flask, and the mixture was stirred in the dark at room temperature for 24 h. Following this, ethylene glycol (2.0 mL) was added to terminate the oxidation reaction. The resulting solution was placed in a dialysis bag (MWCO, 8–14 kDa), dialyzed against distilled water for 3 days to remove residuals, and further freeze-dried for 3 days to obtain the OXG. Subsequently, OXG (10.0 mg) was dissolved in distilled water (1.0 mL). Then 4-arm PEG-NH_2_ (50.0 mg) was added to the solution, and the mixture was allowed to react at room temperature for 5 min to form the XPH.

#### 2.3.3 Construction of ANHD

ANHD was prepared using a similar method to that of XPH, with the addition of CGN into the hydrogel. Specifically, OXG (10.0 mg) was dissolved in distilled water (1.0 mL). CGN (0.5 mg) was then added to the solution, and the mixture was oscillated for 3 min to ensure even dispersion of the CGN. Following this, 4-arm PEG-NH_2_ (50.0 mg) was added to the mixture, and the solution was allowed to react at room temperature for 5 min to obtain the ANHD.

### 2.4 Biomimetic nanozyme activity

#### 2.4.1 Superoxide dismutase (SOD)-like activity

The SOD-like activity was assessed by inhibiting the autooxidation of pyrogallol. Briefly, pyrogallol solution (0.2 mM in HCl, pH 2; 20.0 μL) and CGN solution (20 μg/mL; 500.0 μL) were sequentially added to Tris-HCl buffer (0.1 M, pH 8.2) and mixed. The SOD-like activity was quantified by monitoring the oxidation peak at 320 nm using a UV–vis spectrophotometer.

The ability of CGN to inhibit O_2_^·-^ was assessed using a specific assay kit as per the protocol. The tubes were prepared as follows: blank control (distilled water, 0.05 mL), standard (0.15 mg/mL ascorbic acid solution, 0.05 mL), and test tubes (CGN, XPH, or ANHD solutions at 0.2, 0.4, and 0.8 mg/mL, 0.05 mL). After incubation at 37 °C for 40 min, color reagent (2.0 mL) was added to each tube, and thoroughly mixed. The absorbance was recorded at 550 nm using a Nanodrop One spectrophotometer to calculate the O_2_^·-^ clearance rate.

#### 2.4.2 Catalase (CAT) activity

The dissolved O_2_ concentration was detected using a portable dissolved O_2_ meter to evaluate the CAT-like activity of CGN. The study was divided into three groups: hydrogen peroxide (H_2_O_2_), CGN + H_2_O_2_, and CGN + PBS. For the CGN + H_2_O_2_ group and the CGN + PBS group, 1.0 mg of CGN samples were dispersed in 3.0 mL of 500 mM H_2_O_2_ solution and PBS respectively. Subsequently, the O_2_ concentration was measured using the immersion probe of portable dissolved O_2_ meter. For the H_2_O_2_ group, the O_2_ concentration was directly measured without addition of CGN. The O_2_ concentration in all groups was recorded at 1-min intervals for 5 min.

H_2_O_2_ scavenging activities of CGN, XPH, and ANHD were assessed using a commercial hydrogen peroxide assay kit. Briefly, CGN, XPH, and ANHD samples were dissolved in 1 mM H_2_O_2_ standard working solution (1.0 mL) to prepare test solutions at three concentrations (0.2, 0.4, and 0.8 mg/mL). After incubation for 4 h at 25 ± 1 °C, the reaction systems were established in three parallel groups: (1) blank control (0.25 mL acetone standard+0.075 mL substrate), (2) standard control (0.25 mL H_2_O_2_ standard+0.075 mL substrate), and (3) test groups (0.25 mL H_2_O_2_+CGN, or XPH solutions+0.075 mL substrate). After immediate vortexing and centrifugation (4000×*g*, 10 min), the pellets were resuspended in reaction buffer (0.025 mL), equilibrated for 5 min, and analyzed at 415 nm using a microplate reader to determine residual H_2_O_2_ levels.

#### 2.4.3 Hydroxyl radical (·OH) scavenging activity

The ·OH scavenging activity of the samples was determined using a hydroxyl radical assay kit according to the manufacturer's instructions. The reaction systems were classified into four parallel groups: (1) the blank (0.4 mL distilled water), (2) standard tube (0.2 mL distilled water+0.2 mL 3 % H_2_O_2_), (3) control tube (0.2 mL distilled water+0.2 mL substrate solution), (4) test tubes (0.2 mL 0.2, 0.4, 0.8 mg/mL CGN, XPH or ANHD solutions+0.2 mL substrate solution). The reaction solution (0.4 mL) in each group was added, mixed and incubated at 37 °C for 1 min. The reaction was then immediately terminated and laid out at room temperature for 20 min. The OD value was measured at 550 nm using a spectrophotometer to calculate the ·OH scavenging ability.

### 2.5 Cell experiment

#### 2.5.1 Cell viability

Human corneal epithelial cells (HCECs) and human corneal stromal cells (HCSCs) were seeded in a 96-well plate at a density of 1 × 10^5^ cells per well. The cells were cultured in DMEM/F12 medium supplemented with 1 % penicillin/streptomycin and 10 % FBS. Cells were treated with ANHD at concentrations of 0.1 and 0.01 mg/mL. After 24 h of incubation in a cell incubator, CCK-8 solution (10.0 μL) was added to each well, followed by an additional 1.5-h incubation. The absorbance was measured at 450 nm using a microplate reader, and cell viability was calculated based on the absorbance values.

#### 2.5.2 Live/dead cell staining

HCECs and HCSCs were seeded in 6-well plates at a density of 1 × 10^5^ cells per well. The cells were cultured in a cell incubator for 24 h. In the experimental group, cells were treated with 0.1 and 0.01 mg/mL ANHD (1.0 mL) in culture medium, while the control group received complete medium (1.0 mL). After 24 h of incubation, the medium was removed, and staining solution (0.5 mL), prepared by mixing LiveDye (1.0 μL) and NucleiDye (1.0 μL) per assay buffer (1.0 mL), was added to each well. The cells were incubated for 15 min in the cell incubator, followed by washing two times with PBS. Fluorescent images were captured using a fluorescence microscope.

#### 2.5.3 Scratch assay

HCECs were seeded at a density of 5 × 10^5^ cells per well with complete medium (1.5 mL) and incubated for 24 h in a cell incubator. After incubation, the complete medium was removed, and the cells were washed three times with PBS. To induce serum starvation, 2 % FBS medium was added, and the cells were incubated for an additional 12–24 h. Three evenly spaced scratches were then made across the cell monolayer in the well using a 200-μL pipette tip along the marked lines, and the scratched cells were washed gently three times with PBS to remove any floating cells and debris. The wells were divided into four groups: a control group containing 2 % FBS medium (1.5 mL), and three experimental groups containing the same medium supplemented with CGN, XPH, or ANHD solutions at a concentration of 0.1 mg/mL. Images of the scratch areas were captured at 0 and 12 h using a microscope, and they were further quantitatively analyzed using ImageJ software to assess the cell migration rates.

#### 2.5.4 Intracellular ROS scavenging assay

The ROS scavenging activity of ANHD was evaluated at the cellular level using a ROS detection kit. HCECs and HCSCs were seeded in 6-well plates at a density of 1 × 10^5^ cells per well and cultured overnight at 37 °C in a cell incubator. After the cells adhered to the plate, the medium was removed. Complete medium (1.0 mL), 250 μM H_2_O_2_ solution (1.0 mL), and 0.1 mg/mL ANHD solutions (1.0 mL) were added in the normal, control, and experimental groups, respectively. The cells were incubated at 37 °C for 60 min. Following this, 10 μM DCFH-DA solution (1.0 mL) was added to each well, and the cells were incubated at 37 °C for 5 min. The cells were then washed three times with serum-free medium to remove residual DCFH-DA. Each well was stained with Hoechst nuclear staining solution (1.0 mL). ROS levels were analyzed by flow cytometry (BD FACSCantoTM II). The data were processed using FlowJo v10.8.1.

#### 2.5.5 *In vitro* anti-inflammatory activity

To validate the anti-inflammatory effects of ANHD, RAW 264.7 macrophages were pretreated with lipopolysaccharide (LPS, 100 ng/mL) for 24 h to induce M1 phenotype polarization. Then 0.1 mg/mL ANHD and PBS were added to the cells, respectively, and incubated for another 24 h. The cells were harvested, washed three times with PBS, and stained with anti-CD86 and anti-CD206 antibodies (4 °C, 60 min). After staining, the cells were centrifuged at 300×*g* for 5 min, resuspended in FACS buffer (500.0 μL), and analyzed by flow cytometry (BD FACSCanto™ II). The data were processed using FlowJo v10.8.1.

### 2.6 *In vitro* antibacterial test

#### 2.6.1 CFU assay

The *Staphylococcus epidermidis* used in the study was clinically isolated from infected patients at Qingdao Eye Hospital of Shandong First Medical University, China. All strains were cultured in Luria-Bertani (LB) medium at 37 °C prior to use. A single bacterial colony was inoculated into LB medium (5.0 mL) and cultured overnight at 37 °C. The bacterial suspension was aliquoted into centrifuge tubes, each containing suspension (1.0 mL) at a concentration of 1 × 10^8^ CFU/mL. The suspensions were centrifuged at 6000 rpm for 5 min, and the supernatant was discarded. The bacterial pellets were resuspended in PBS (1.0 mL), 1 mg/mL sample solution (CGN, XPH, ANHD, 1.0 mL), or 1 mg/mL gatifloxacin solution (1.0 mL), corresponding to the control group, experimental group, and positive control group, respectively. The suspension samples were incubated at 37 °C for 2 h and serially diluted to a final concentration of 1 × 10^3^ CFU/mL. The diluted suspension (50.0 μL) was inoculated onto LB plates, and the plates were incubated at 37 °C for 24 h, after which photographs were taken, and the colonies were counted.

#### 2.6.2 Live/dead staining assay

The antibacterial activity of ANHD was assessed using a live/dead bacterial viability kit. Bacterial suspension (1 × 10^8^ CFU/mL, 1.0 mL per tube) was aliquoted into 2-mL centrifuge tubes, and the supernatant was removed by centrifugation. The bacterial pellets were resuspended in PBS, XPH, CGN, or ANHD solution (1.0 mL). The samples were incubated at 37 °C for 24 h, followed by staining with the live/dead bacterial viability kit. Live bacteria exhibited green fluorescence, while dead bacteria showed red fluorescence. Fluorescent images were captured using a fluorescence microscope, and the fluorescence intensity was quantitatively analyzed using ImageJ software.

#### 2.6.3 Bacterial morphology observation

SEM was used to evaluate the morphological changes of *S. epidermidis* after different treatments. In detail, 1 mg/mL ANHD, gatifloxacin, or levofloxacin solution (500.0 μL) was mixed with a 1 × 10^8^ CFU/mL bacterial suspension (500.0 μL) in a centrifuge tube. As a control, PBS (500.0 μL) was mixed with the same bacterial suspension (500.0 μL) in another centrifuge tube. The samples were incubated overnight at 37 °C. After centrifugation at 10,000 rpm for 10 min, the supernatant was discarded, and the bacterial pellet was collected. The pellet was washed three times with PBS, subsequently fixed overnight in the electron microscope fixative, and finally centrifuged to remove the supernatant. The samples were dehydrated using 30 %, 50 %, 70 %, and 100 % ethanol solutions (v/v), with centrifugation after each step to remove the supernatant. The dehydrated bacterial samples were then placed on pre-cleaned silicon wafers and air-dried overnight in a clean bench. Finally, SEM was used to observe and document the morphological images of the bacterial samples.

#### 2.6.4 Inhibition of biofilm formation

The anti-biofilm activity of ANHD was evaluated using crystal violet (CV) staining. In brief, ANHD, gatifloxacin, or levofloxacin (1.0 mg/mL, 100.0 μL), TSB medium (95.0 μL), and bacterial suspension (2 × 10^9^ CFU/mL, 5.0 μL) were added to each well in a 96-well plate. The samples were incubated at 37 °C for 48 h, and then washed with PBS to remove planktonic bacteria. Next, the samples were fixed with methanol for 15 min, air-dried, and the remaining biofilms were stained with 1 % CV solution for 20 min. The wells were then washed three times with PBS to remove the excess CV, and the samples in the wells were air-dried. The attached CV was dissolved in acetic acid, and the absorbance was measured at 590 nm using a microplate reader to quantitatively analyze the biofilm.

### 2.7 Validation of hybrid cross-linked cornea

#### 2.7.1 Biomechanical evaluation

The mice (female, *n* = 20) were anesthetized *via* intraperitoneal injection of 0.6 % pentobarbital sodium, followed by intrastromal injection of ANHD (1.0 μL) using a microsyringe. At predetermined time points (3, 6, 12, 24, 72, and 168 h), the mice were euthanized by cervical dislocation. Corneas were immediately harvested and embedded at −80 °C in Tissue-Tek® O.C.T. compound (Sakura Finetek, Netherlands). Sagittal sections (100 μm thickness) were prepared using a Leica CM1950 cryostat (Germany) and mounted on adhesive slides (Citotest, China). For biomechanical testing, sections were secured in Petri dishes with PBS and fully hydrated during nanoindentation using a Piuma system (OPTICS11, Netherlands) equipped with a spherical tip (24.5 μm radius, 0.59 N/m stiffness). The testing protocol consisted of 10 μm indentations from the epithelial surface, including 2 s loading, 1 s hold, and unloading phases to determine elastic modulus. Each sample was measured three times, and the average value was taken for statistical analysis.

#### 2.7.2 Antibacterial assay of *ex vivo* cornea

The mice (female, *n* = 20) were anesthetized *via* intraperitoneal injection of 0.6 % pentobarbital sodium. ANHD (1.0 μL) was injected into the corneal stroma using a microsyringe. The mice were euthanized at 6 h post-injection and the hybrid crosslinked corneas were immediately excised and stored at −80 °C. The corneas were incubated in bacterial suspension (200.0 μL, 1 × 10^7^ CFU/mL) for 4 h. The samples (50.0 μL) were serially diluted, spread on LB agar plates (6 cm diameter), and aerobically incubated at 37 °C for 24 h. CFUs were quantified using an automated colony counter, with documentation *via* digital imaging.

#### 2.7.3 *In vivo* drug sustained release

The *in vivo* release profile of gatifloxacin from ANHD was investigated by determining the gatifloxacin concentration in aqueous humor from New Zealand White rabbits. In detail, rabbits were anesthetized *via* intraperitoneal injection of 3 % pentobarbital sodium (50.0 mg/kg) and topical 0.5 % proparacaine hydrochloride. A microsyringe was then used for intrastromal injection of ANHD (200.0 μL). Aqueous humor (50.0 μL) was aspirated from the anterior chamber of rabbit eyes at predetermined time points (3, 6, 12, 24, 48, 96, 144, and 192 h) and mixed with an equal volume of methanol. The mixture was centrifuged at 15,000 rpm for 10 min, and the supernatant was collected for quantitative analysis of gatifloxacin using ultra-performance liquid chromatography-tandem mass spectrometry (UPLC-MS/MS).

#### 2.7.4 Hydrogel degradation evaluation

*In vitro* degradability of the hydrogel was investigated by incubating lyophilized ANHD sample (46.0 ± 0.2 mg) in a PBS solution (pH 6 and 7, 10.0 mL). Samples were retrieved daily for six days, rinsed with distilled water, freeze-dried for 24 h, and weighed to calculate the degradation rate of hydrogel at each day.

### 2.8 Animal experiments

C57BL/6 mice (female, 6–8 weeks old) were purchased from Shandong Taike Biotechnology Co., Ltd. (China). All animal experiments were conducted in strict accordance with ARVO guidelines and were approved by the Medical Ethics Committee of Eye Institute of Shandong First Medical University, China (SDSYKYJS No.20240219).

#### 2.8.1 BK model

The *S. epidermidis* keratitis model was adopted to evaluate the *in vivo* antibacterial activity of ANHD. The mice (*n* = 60) were anesthetized *via* intraperitoneal injection of 0.6 % sodium pentobarbital, and the bacterial suspension (1 × 10^7^ CFU/mL, 2.0 μL) was injected into the corneal stroma using a microsyringe. After 12 h, the mice were re-anesthetized, and randomly divided into four groups, which were given PBS, ANHD, gatifloxacin, and levofloxacin solutions, respectively. On days 1, 3, 5, and 7, slit-lamp microscopy was used to capture eye images, and the severity was scored based on standard evaluation criteria. Central corneal thickness was measured using anterior segment optical coherence tomography (AS-OCT), and intraocular pressure (IOP) was assessed with a Tonolab animal tonometer. Sterile swabs were used to collect the secretions on the ocular surface, which were transferred into centrifuge tubes containing PBS (1.0 mL) and thoroughly mixed. An aliquot (50.0 μL) from the centrifuge tube was plated onto LB agar plates and incubated at 37 °C for 24 h to observe and photograph the bacterial colonies for statistical analysis. The ROS levels in corneal tissues were evaluated by DHE staining on day 7. On days 1, 3, 5, and 7, three mice per group were sacrificed, and their corneas were collected for mechanical testing. IL-1β, IL-6, and TNF-α of the collected corneas on day 7 were analyzed *via* qRT-PCR and ELISA. In addition, the corneas were further collected for flow cytometry analysis and hematoxylin and eosin (H&E) staining.

#### 2.8.2 *In vivo* biocompatibility assessment

The biosafety of CGN, XPH, or ANHD was evaluated by injection of each solution (1.0 μL) into the corneal stroma of mice (*n* = 20) using a microsyringe. On days 1, 3, 5, and 7, IOP and central corneal thickness were measured. On day 7, 1 % fluorescein sodium (10.0 μL) was applied to the ocular surface, and slit-lamp microscopy was used to examine the eye condition under cobalt blue light. After observation, the mice were euthanized, and their eyeballs, along with heart, liver, spleen, lungs, and kidneys, were collected. The tissues were rinsed in saline, fixed in 4 % paraformaldehyde, embedded in paraffin, and subjected to H&E staining for histopathological analysis.

#### 2.8.3 Corneal injury healing assay

Mice were anesthetized *via* intraperitoneal injection of 0.6 % sodium pentobarbital. A 2.5-mm trephine was used to mark the corneal epithelium at the center of each mouse cornea. Topical anesthesia was administered using 0.5 % proparacaine eye drops, and the marked corneal epithelium was removed with an Algerbrush® II corneal epithelium scraper. The mice were divided into four groups: PBS, CGN, XPH, and ANHD groups. The corresponding solutions were administered by eye drop twice a day, with 10.0 μL per drop. At specific time intervals (0, 12, 24, 36, and 48 h), fluorescein solution was applied to the ocular surface, and corneal images were captured using a digital microscope under cobalt blue light. The epithelial repair area was quantitatively analyzed using ImageJ software.

#### 2.8.4 Neuroregeneration study

The mice were administered with PBS, CGN, XPH, and ANHD for seven days after the removal of corneal epithelium. Subsequently, the mouse eyeballs were collected and fixed in 4 % paraformaldehyde for 15 min. The corneas were then dissected in a PBS solution, and fixed in 4 % paraformaldehyde for an additional 2 h, before being washed three times with PBS. Under a microscope, the corneas were carefully cut into four flaps that remained connected at the base. The samples were placed in a blocking solution containing 0.3 % Triton X-100 and 3 % bovine serum albumin solution for 2 h at room temperature. Then they were transferred to a 96-well plate, and a tubulin-488 antibody diluted 1:300 in PBS was added to each well. The samples were incubated at room temperature for 3 h. After incubation, the samples were washed three times with PBST solution (0.3 % Triton X-100 and 0.05 % Tween-20 in PBS). Finally, the samples were mounted using an anti-fading mounting medium and imaged using a confocal microscope to assess corneal nerve regeneration.

### 2.9 Statistical analysis

All experiments were performed at least three times to ensure data reproducibility. The data were presented as mean ± standard deviation. Statistical analyses, including Student's *t*-test and one-way ANOVA, were conducted using Prism 9.0 software (GraphPad, San Diego, CA, USA). A *p*-value of less than 0.05 was considered statistically significant for intergroup comparisons.

## 3 Results and discussion

### 3.1 Synthesis and characterizations

The conventional CXL was accomplished by the penetration of riboflavin into corneal stroma after epithelial removal to induce photochemical cross-linking between stromal amino and carboxyl groups, thereby enhancing corneal mechanical strength. Inspired by this, a Schiff base chemistry-based cross-linking strategy was developed by the reaction of aldehyde groups in oxidized bacterial polysaccharides with amino groups in 4-arm PEG-NH_2_ to reinforce the corneas, coupled with inherent pro-healing properties. Here, the acetylation degree of xanthan gum was determined to be 2.9 % through titration experiments, and aldehyde content in oxidized xanthan gum (%) was estimated to be 2.3 % using the acid-base titration technique with hydroxylamine hydrochloride. The molecular weight of xanthan gum was determined to be approximately 362 kDa using gel permeation chromatography (Fig. S1, supporting information). Given the burst of ROS in the lesion area of BK, ultra-small cerium nanoparticles were engineered to support the ROS clearing activity of nanozyme. Besides, the ROS-clearing ability of cerium nanozymes was further integrated with the antibacterial function of antibiotics by the reaction of cerium nitrate and gatifloxacin. Ultimately, a nanozyme hydrogel platform with corneal reinforcement, antibacterial, pro-healing, and ROS-clearing activities was designed for the treatment of BK.

**Fig. 1:**
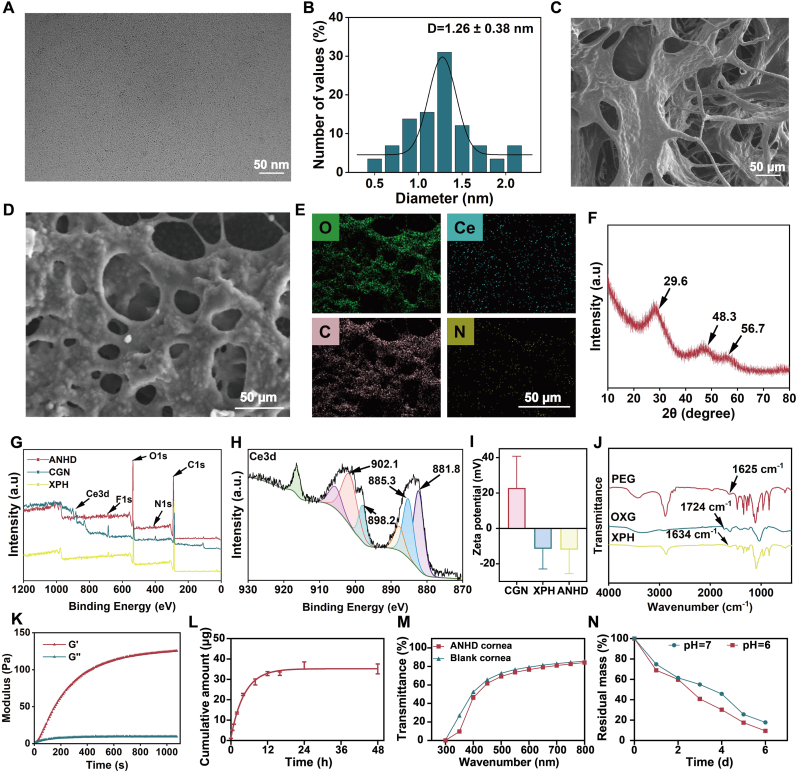
Physicochemical characterization. (A) TEM image of CGN. (B) Particle size distribution of CGN. SEM images of (C) XPH and (D) ANHD. (E) EDS mapping of ANHD. (F) XRD spectra of CGN. (G) XPS spectra of CGN, XPH, and ANHD. (H) Deconvoluted XPS spectra of Ce 3d in the CGN. (I) Zeta potential of CGN, XPH, and ANHD. (J) FT-IR spectra of 4-arm PEG-NH_2_, OXG, and XPH. (K) Storage moduli (G′) and loss moduli (G″) of XPH. (L) *In vitro* gatifloxacin release from ANHD. (M) Light transmittance of ANHD at different wavelengths. (N) Degradation curve of ANHD.

The morphological characteristics of CGN was observed by TEM. The images showed that the average particle size of CGN was 1.26 ± 0.38 nm (Fig. 1A and B). Fig. S2 (supporting information) exhibited the optical photograph of XPH, which showed the gel-like state of hydrogel. SEM confirmed the macroporous architecture of XPH and ANHD (Fig. 1C and D). Mapping analysis revealed the uniform distribution of Ce, C, O, and N elements in ANHD, indicating successful CGN incorporation (Fig. 1E). XRD analysis revealed the crystal structure of CGN (Fig. 1F). In the XRD pattern of CGN, the diffraction peaks at 29.6, 48.3, and 56.7 corresponded to those of CeO_2_ nanoparticles. XPS analysis confirmed the presence of C, O, N in XPH, C, N, O, F in CGN, C, N, O and F in ANHD (Fig. 1G and H). Ce^3+^and Ce^4+^ oxidation states in CGN were confirmed by the high resolution XPS scan of Ce 3d. In the Ce 3d spectra of CGN, the peaks at 885.3, 902.1, 881.8, and 898.2 eV can be ascribed to Ce^4+^ 3d5/2, Ce^4+^ 3d3/2 peaks, Ce^3+^ 3d5/2 and Ce^3+^ 3d3/2 peaks, respectively. The presence of Ce^3+^and Ce^4+^ contributed to the SOD and CAT activities of CGN. Zeta potential measurement indicated that the surface charges of CGN, XPH and ANHD were +22.8, −11.5, and −12.0 mV, respectively (Fig. 1I). The high charge of CGN demonstrated the stability of the nanozyme. The negatively charged hydrogel crosslinked in the corneal stroma might generate electrostatic repulsion against bacterial surfaces, thereby inhibiting the microbial adhesion. FT-IR spectra were used for the structural identification of Schiff base hydrogels (Fig. 1J). The absent peak signals of bending vibration of amine in the 4-arm PEG-NH_2_ at 1625 cm^−1^ and stretching vibration of carbonyl in the OXG at 1724 cm^−1^ as well as the presence of stretching vibration of Schiff base in the hydrogel at 1634 cm^−1^ indicated the successful preparation of hydrogel by the Schiff base reaction between amine in 4-arm PEG-NH_2_ and carbonyl groups in the OXG.

The rheological property of the XPH was evaluated with a rotational rheometer (Fig. 1K). The hydrogel presented the intersection points of lost modulus (G″) and stored modulus (G′) within a short time, indicating a rapid gelation rate during the formation of XPH. The *in vitro* drug release profile of gatifloxacin was quantitatively analyzed using UPLC (Fig. 1L, Fig. S3, supporting information). The cumulative release curve demonstrated an initial rapid release within the first 9 h and a sustained release that reached a plateau by 24 h. These results highlighted the capability of ANHD for delivery of gatifloxacin. Fig. 1M presented the optical performance of ANHD. Compared with the normal mouse corneas, the hydrogel crosslinked corneas exhibited similar light transmittance, indicating that *in situ* Schiff base crosslinking maintained excellent optical transparency without compromising visual function. *In vitro* degradability of ANHD was assessed by calculating the mass ratio of residual hydrogel to total hydrogel. As shown in Fig. 1N, mass percentages of ANHD decreased to 60.0 % ± 2.1 %, 30.2 % ± 1.4 % and 9.5 % ± 1.9 % (pH = 6) at 2, 4, and 6 days, respectively, after immersion into the PBS solution, indicating the good *in vitro* degradability of XPH. In addition, the swelling ratios of XPH after immersion into PBS for both 6 and 12 h were all more than 1100 %, compared with the dried XPH (Fig. S4, supporting information). Rheological characterization revealed that, in comparison with XPH, ANHD exhibited typical shear-thinning behavior. Specifically, the viscosity of ANHD decreased with increasing shear rate and subsequently reached a plateau, which was consistent with the characteristic behavior of non-Newtonian fluids (Fig. S5, supporting information).

### 3.2 Biocompatibility evaluation

**Fig. 2:**
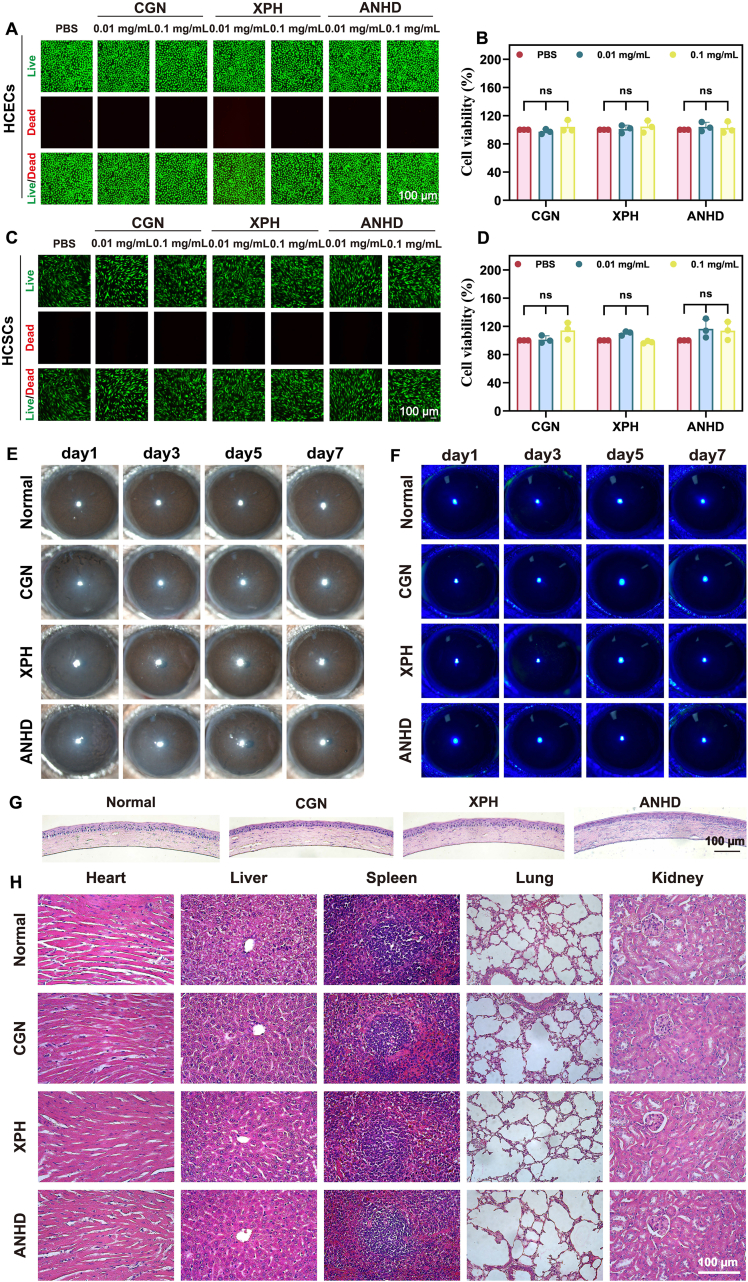
Biocompatibility assessment of ANHD. Live/dead cell staining images and CCK-8 viability of (A, B) HCECs and (C, D) HCSCs after incubation with CGN, XPH, and ANHD solutions (0.01 and 0.1 mg/mL) for 24 h. Scale bar, 100 μm. (E) Slit-lamp images and (F) fluorescein staining of mouse eyes post-treatment by CGN, XPH, and ANHD on days 1, 3, 5, and 7. (G) H&E-stained corneal sections post-treatment by CGN, XPH, and ANHD (scale bar, 100 μm; *n* = 3). (H) Visceral histopathology of heart, liver, spleen, lung, and kidney (scale bar, 100 μm; *n* = 3).

The influence of the CGN, XPH, and ANHD on the cell viability of HCECs and HCSCs was assessed using live/dead cell staining and the CCK8 assay. The staining results showed that there was significant green fluorescence with minimal red fluorescence in the PBS, CGN, XPH, and ANHD groups (0.01 and 0.1 mg/mL), indicating no cytotoxicity of CGN or XPH (0.01 and 0.1 mg/mL) to HCECs and HCSCs (Fig. 2A and C). The CCK-8 results showed that the cell viabilities of HCECs and HCSCs in the CGN, XPH, and ANHD groups (0.01 and 0.1 mg/mL) were all more than 94 %. No significant differences were observed in the cell viabilities of HCECs and HCSCs between the PBS and the CGN, XPH or ANHD groups (*p* > 0.05) (Fig. 2B and D).

The *in vivo* biocompatibility of CGN, XPH, and ANHD was evaluated by murine intrastromal injections (0.5 mg/mL, 1.0 μL), followed by slit-lamp imaging, corneal thickness measurement, IOP assessment, and H&E staining. The slit lamp observation showed that the corneas in all groups except the normal group appeared slightly turbid on the first day. The turbidity gradually decreased on the 3rd, 5th and 7th day. On the 7th day, there was no significant difference between the normal and treatment groups (Fig. 2E). Fluorescein staining showed that there were no obvious corneal epithelial defects in mice from any group (Fig. 2F). In addition, there was no significant difference among four groups in the central corneal thickness and IOP (Fig. S6, supporting information). Consistently, H&E staining demonstrated no corneal edema or inflammatory infiltration (Fig. 2G), and major organs (heart, liver, spleen, lungs, and kidneys) showed no pathological alterations (Fig. 2H).

The biocompatibility of ANHD was further validated using hemolysis tests in New Zealand White rabbits. While 0.1 % Triton X-100 (positive control) caused complete hemolysis, neither 0.1 % BSA (negative control) nor 0.1 % ANHD affected erythrocyte integrity. In addition, the blood was collected from the vein at the ear margin for complete blood cell count analysis 12 h post injection of ANHD into the corneal stroma of rabbit eyes. Compared with the PBS group, there was no significant difference in hematological parameters such as white blood cells, red blood cells and platelets in the ANHD group (Fig. S7, supporting information). All these results confirmed the biocompatibility of ANHD.

### 3.3 Antioxidant activity

**Fig. 3:**
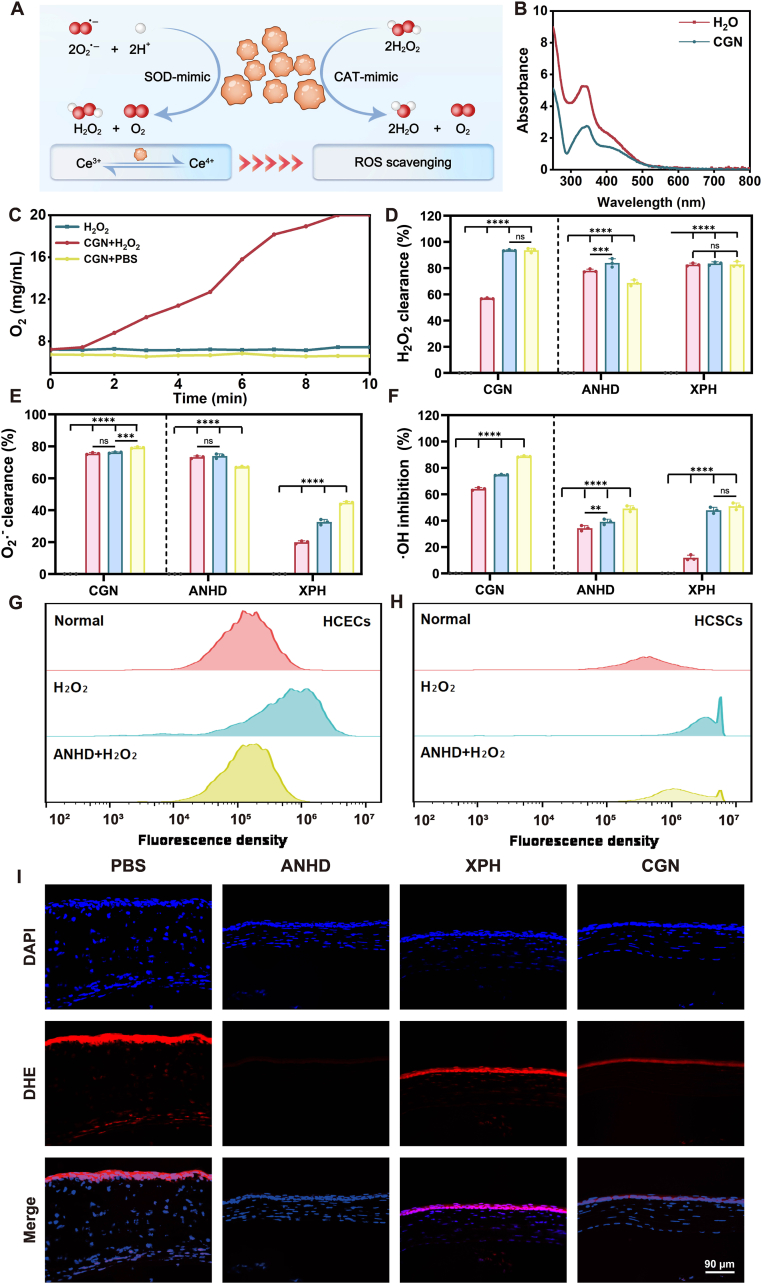
Antioxidant activity assessment. (A) SOD/CAT activities of CGN. (B) The absorption spectra of pyrogallol autoxidation system without and with CGN. (C) O_2_ generation capacity assessment. (D–F) The ability of CGN, XPH, and ANHD to clear H_2_O_2_, ·OH, and O_2_^•−^. Flow cytometric analysis of ROS levels in (G) HCECs and (H) HCSCs post-treatment with H_2_O_2_ and H_2_O_2_+ANHD. (I) Representative DHE immunofluorescence staining images of BK corneas on day 7 post-treatment with CGN, XPH, and ANHD. DAPI (nuclei, blue), DHE (ROS, red).

The antioxidant capacity of CGN was assessed through its intrinsic SOD- and CAT-mimetic activities (Fig. 3A). SOD-like activity was evaluated using the catechol autooxidation assay, where the absence of CGN resulted in a characteristic peak at 320 nm, indicative of O_2_^·-^ accumulation. Notably, addition of CGN demonstrated significant inhibition of the autooxidation process (Fig. 3B), confirming the potent SOD-mimetic property of CGN. O_2_ generation was monitored to assess the CAT-like activity. Time-dependent O_2_ evolution was observed in the CGN + H_2_O_2_ system (Fig. 3C), demonstrating the ability of CGN to decompose H_2_O_2_. In addition, XPH is prepared by the reaction of antioxidant OXG with PEG (Fig. S8, supporting information). The antioxidant capacities of CGN, XPH, and ANHD were further evaluated by investigating their scavenging capabilities on O_2_^·-^, H_2_O_2_, and ·OH using the corresponding colorimetric assay kits (Fig. 3D–F). CGN demonstrated concentration-dependent ·OH scavenging activity, with clearance rates increasing from 64.1 % at 0.2 mg/mL to 74.7 % at 0.4 mg/mL and 88.6 % at 0.8 mg/mL. In contrast, H_2_O_2_ elimination exhibited the efficiency of 56.8 % at 0.2 mg/mL and 93.6 % at both 0.4 and 0.8 mg/mL. Meanwhile, O_2_^·-^ removal consistently maintained high efficacy, exceeding 75.4 % at all tested concentrations. ANHD showed superior performance in O_2_^·-^ clearance compared to XPH at all concentrations, while its H_2_O_2_ clearance and ·OH scavenging capacities were comparable to XPH. In addition, we further compared the H_2_O_2_ clearance rate of CGN at pH 5, 6, and 7. The results revealed that there was no significant difference among H_2_O_2_ clearance rates under these pH conditions, suggesting the pH stability of CGN (Fig. S9, supporting information).

The ROS scavenging capacity of ANHD at the cellular level was evaluated by flow cytometry analysis of HCECs and HCSCs. The results showed minimal ROS-stained cells in the normal group (Fig. 3G and H). In contrast, H_2_O_2_ stimulation significantly increased the intensity of ROS staining, indicating robust oxidative stress activation (Fig. 3G). Notably, the addition of ANHD attenuated the H_2_O_2_ induced increase, demonstrating its potent capacity to suppress oxidative stress (Fig. 3H).

The *in vivo* ROS scavenging capacity of ANHD was evaluated through corneal DHE staining from PBS, ANHD, CGN, and XPH treated groups, as illustrated in Fig. 3I. The ROS level was quantitatively analyzed by measuring the intensity of green fluorescence. The results revealed distinct ROS scavenging capacities among different treatment groups. The ANHD-treated group exhibited the weakest fluorescence signal, demonstrating the most significant ROS suppression. CGN displayed moderate scavenging activity, with fluorescence intensities following ANHD in descending order. In addition, XPH treatment showed lower ROS levels than the PBS group but the higher levels than those in the CGN and ANHD groups. The superior antioxidant performance of ANHD might be attributed to synergistic antioxidant effects of CGN and XPH.

### 3.4 Anti-inflammatory effects

**Fig. 4:**
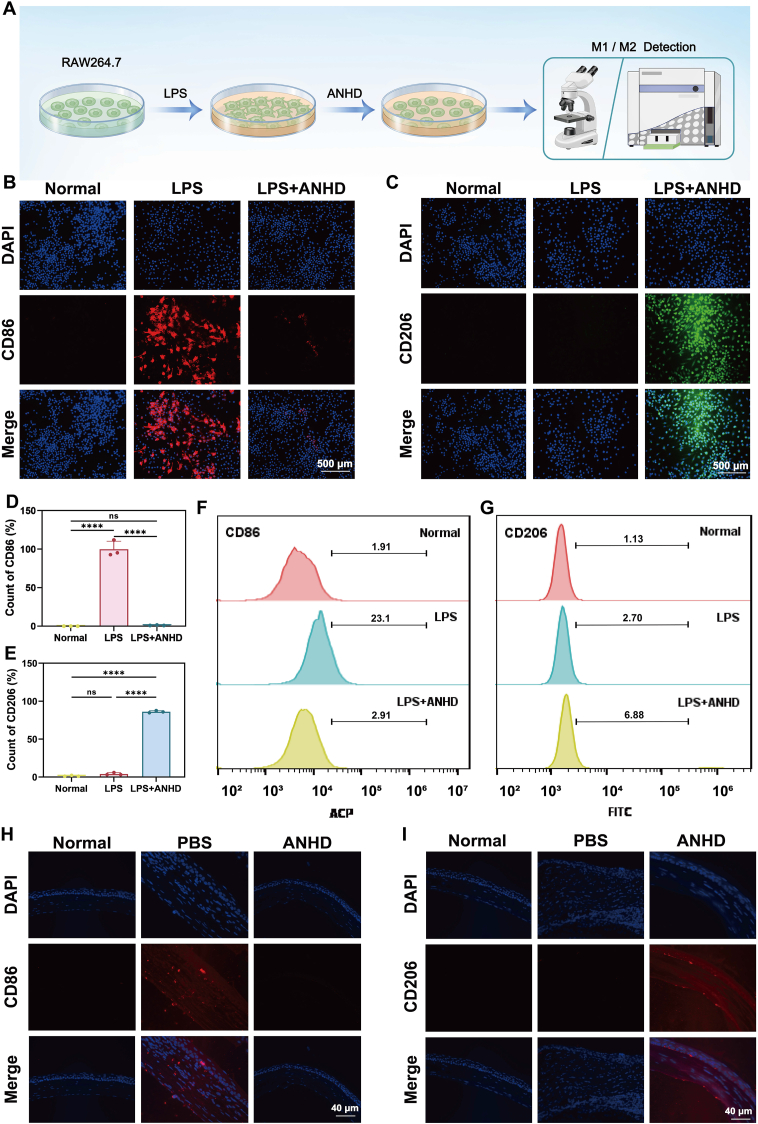
Anti-inflammatory activity evaluation. (A) Schematic representation of the *in vitro* anti-inflammatory assay. CD86 and CD206 expression in RAW 264.7 macrophages through (B–E) immunofluorescence and (F, G) flow cytometry analysis of corneas from the normal, LPS-treated, and LPS + ANHD groups. Expression levels of (H) CD86 and (I) CD206 in BK corneas by immunofluorescence from the normal, PBS, and ANHD groups (*n* = 3).

LPS-stimulated RAW 264.7 macrophages were used to assess the anti-inflammatory activity of ANHD at the cellular level by immunofluorescence staining, flow cytometry analysis and qRT-PCR (Fig. 4A). Immunofluorescence staining showed that the LPS-stimulated cells exhibited high expression of CD86 and low expression of CD206, while ANHD treatment significantly inhibited the expression of CD86 and upregulated the expression of CD206 (Fig. 4B and C). Quantitative results revealed that ANHD treatment significantly reduced the proportion of M1 macrophages (CD86^+^) from 23.1 % to 2.9 % and markedly increased expression of the M2 marker (CD206+) from 2.7 % to 6.9 % compared to LPS-treated groups, demonstrating the role of ANHD in suppressing M1 polarization and alleviating LPS-triggered inflammation (Fig. 4D and E). Meanwhile, flow cytometry analysis showed the similar results as the immunofluorescence staining analysis (Fig. 4F and G). In addition, after 24 h of culture, obvious morphological differences were observed among the three treatment groups. Cells treated with LPS presented a slender spindle-shaped morphology, which was a characteristic of M1-polarized macrophages. In contrast, the LPS + ANHD group mainly maintained a round cell morphology, with some short spindle-shaped cells, which was consistent with M2-like macrophages (Fig. S10, supporting information). In addition, qRT-PCR analysis showed significant downregulation of IL-1β, IL-6, and TNF-α levels in the ANHD-treated corneas compared to the PBS group (Fig. S11, supporting information). The *in vivo* anti-inflammatory effect of ANHD was further evaluated by corneal immunofluorescence. The PBS-treated corneas showed strong CD86 staining and weak CD206 signal, reflecting an inflammatory response. In contrast, ANHD treatment reversed the polarization pattern (Fig. 4H and I). Collectively, these results demonstrated potent anti-inflammatory effects of ANHD both *in vitro* and *in vivo*.

### 3.5 *In vitro* antibacterial effects

**Fig. 5:**
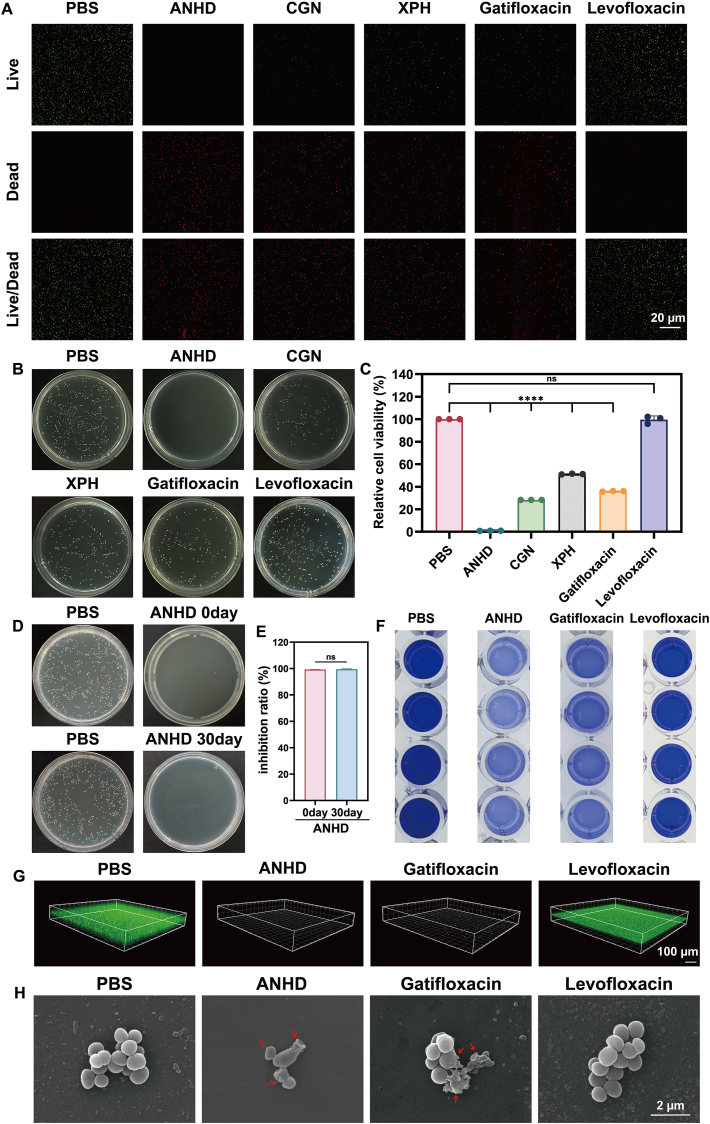
*In vitro* antibacterial activity evaluation. (A) Live/dead bacterial staining of corneas in six treatment groups (scale bar, 20 μm). (B) CFU counts of MDR *S. epidermidis* after treatment with PBS, ANHD, CGN, XPH, gatifloxacin, or levofloxacin (1 mg/mL). (C) Quantitative analysis of bacterial survivals. (D) Antibacterial activity of ANHD after storage at 4 °C for 30 days and (E) quantitative analysis of bacterial survivals. (F) CV staining assay, (G) CLSM images of biofilms, (H) SEM images of *S. epidermidis* after treatment with PBS, ANHD, gatifloxacin, and levofloxacin.

The *in vitro* antibacterial capacities of CGN, XPH, and ANHD were validated by live/dead bacterial staining, CFU analysis, inhibition of biofilm, and SEM. In contrast to intense green signals (viable cells) in the PBS and levofloxacin groups, dominant red fluorescence (dead cells) was present in CGN, gatifloxacin, and XPH groups. Especially, there were nearly no obvious green signals in the ANHD group (Fig. 5A). These results suggested the possible synergistic antibacterial effects between CGN and XPH in ANHD. The CFU results were in accordance with those in live/dead bacterial staining. As shown in Fig. 5B and C, there were no significant differences in bacterial counts between PBS and levofloxacin groups, whereas CGN, XPH, and gatifloxacin reduced bacterial survival to 28 %, 51 %, and 36 %, respectively. Notably, antibacterial efficiency was more than 99 % in the ANHD group. We further investigated the antibacterial efficacy of ANHD after storage in a 4 °C refrigerator for 30 days to assess its stability (Fig. 5D and E). The results revealed the comparable antibacterial activity of ANHD on day 30 to that of freshly prepared solution.

The inhibitory effect of ANHD on biofilm formation was assessed using CV assays. Quantitative analysis of the biofilms in the four groups was conducted *via* CV staining (Fig. 5F, Fig. S12, supporting information). Compared to the PBS group (3.75 ± 0.10), ANHD showed a significantly reduced peak absorbance at 590 nm (0.56 ± 0.01). Gatifloxacin treatment (0.98 ± 0.25) exhibited moderate inhibition of biofilm formation but was less effective than ANHD. The levofloxacin group (3.71 ± 0.12) showed no significant difference compared to the control group. These results confirmed the robust inhibitory activity of ANHD against *S. epidermidis* biofilm formation. Further confocal laser scanning microscopy (CLSM) examination after SYTO 9/PI staining revealed strong green fluorescence in the PBS group, indicating well-established biofilms (Fig. 5G). In contrast, both the ANHD and gatifloxacin groups displayed faint green fluorescent spots, with ANHD showing the weakest intensity, thus confirming its superior bactericidal efficacy and substantial suppression of *S. epidermidis* biofilm formation. SEM images were collected to evaluate the effect of ANHD on the morphology of *S. epidermidis* (Fig. 5H). The PBS and levofloxacin-treated bacteria retained smooth and intact surfaces, whereas surface roughness and cellular collapse (arrows) were present in the gatifloxacin- and ANHD-treated specimens. These observations confirmed the superior bactericidal efficacy of ANHD over both levofloxacin and gatifloxacin.

CGN, as an ultrasmall nanozyme, possessed a high surface area-to-volume ratio that exposes catalytic active sites. The characteristic facilitated ROS-clearing and antibacterial activities within the corneal tissue, while the ultrasmall size also allowed for eventual metabolic clearance. The CGN was uniformly dispersed and stable in the XPH crosslinked corneal stroma, ensuring uniform dispersion and preventing aggregation.

### 3.6 Corneal reinforcement and repair

**Fig. 6:**
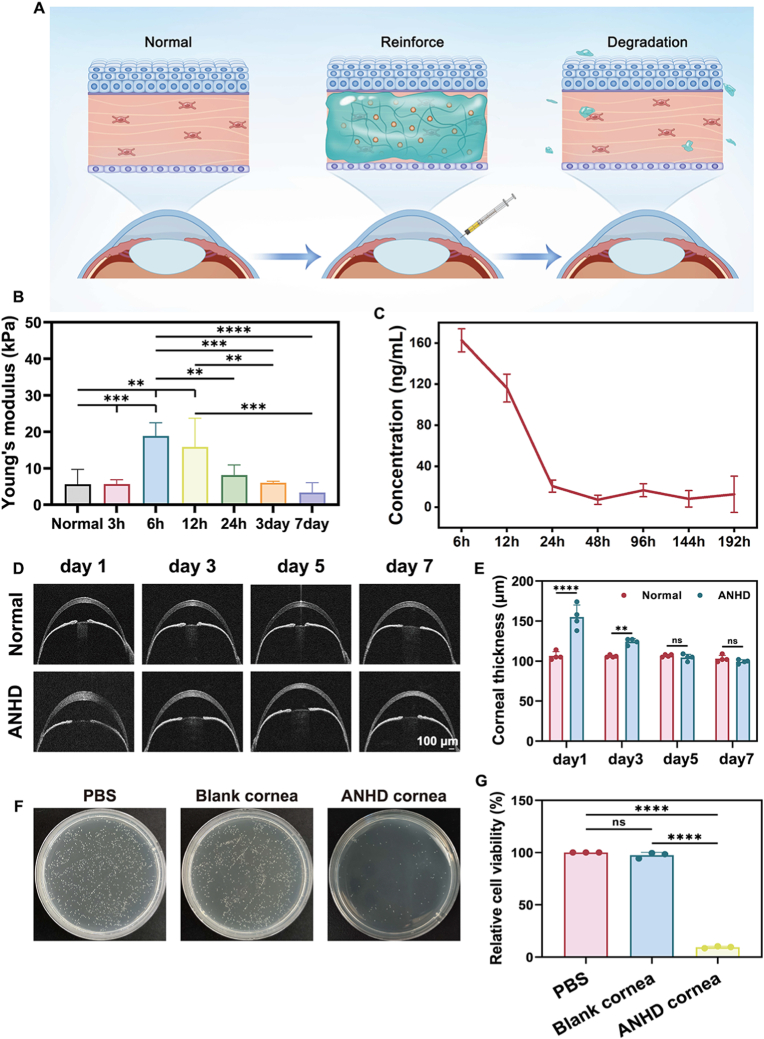
Characterization of *in situ* corneal crosslinking. (A) Pattern diagram of corneal crosslinking. (B) Time-course changes in murine corneal Young's modulus following ANHD administration. (C) *In vivo* gatifloxacin release curve plotted by determination of its concentration in the aqueous humor of rabbit eyes after *in situ* corneal crosslinking. (D) AS-OCT images of corneas after *in situ* corneal crosslinking and (E) quantification of corneal thickness on days 1, 3, 5, and 7. (F) CFU counts of *S. epidermidis* after treatment with PBS, blank cornea, and ANHD crosslinked cornea, and (G) quantitative analysis of bacterial survivals.

Corneal stromal crosslinking was characterized by evaluating corneal biomechanical strength, gatifloxacin release, corneal stromal thickness, and antibacterial performance using the *ex vivo* murine corneas after *in vivo* intrastromal injection of ANHD (Fig. 6A). Corneal biomechanical strength was dynamically assessed by measuring the Young's modulus. Modulus values exhibited an initial increase from 3 to 12 h post-treatment, followed by a gradual return to normal levels by day 7, indicating the capacity of ANHD to induce potent yet reversible mechanical enhancement (Fig. 6B). Pharmacokinetic analysis was conducted using LC-MS/MS by detecting gatifloxacin concentration in aqueous humor over 8 days (Fig. 6C, Fig. S13, supporting information). Gatifloxacin displayed rapid initial clearance from 158.5 ng/mL at 6 h to 21.5 ng/mL by 24 h, reaching a nadir of 5.9 ng/mL by day 8. The pharmacokinetic profile suggested the therapeutic strategy provided immediate antibacterial activity.

Central stromal thickness following intrastromal injection was monitored *via* AS-OCT. Imaging revealed a significant increase in stromal thickness of mice in the ANHD group on day 1 post-injection (155.5 ± 15.1 μm) compared to that in the normal group (106.5 ± 4.5 μm; Fig. 6D and E). Stromal thickness decreased to 124.3 ± 3.8 μm by day 3, and returned to normal levels (104.5 ± 4.1 μm *vs* 106.8 ± 1.3 μm) by day 5 post-injection, demonstrating the good biodegradation of ANHD. Corneal antibacterial efficacy of ANHD was assessed using CFU analysis. The results revealed no statistical difference of bacterial survival rates between the PBS (100.0 %) and XPH groups (97.4 % ± 2.8 %) (Fig. 6F and G). ANHD treatment reduced bacterial survival to 9.4 % ± 0.9 %, indicating its good antibacterial capacity.

**Fig. 7:**
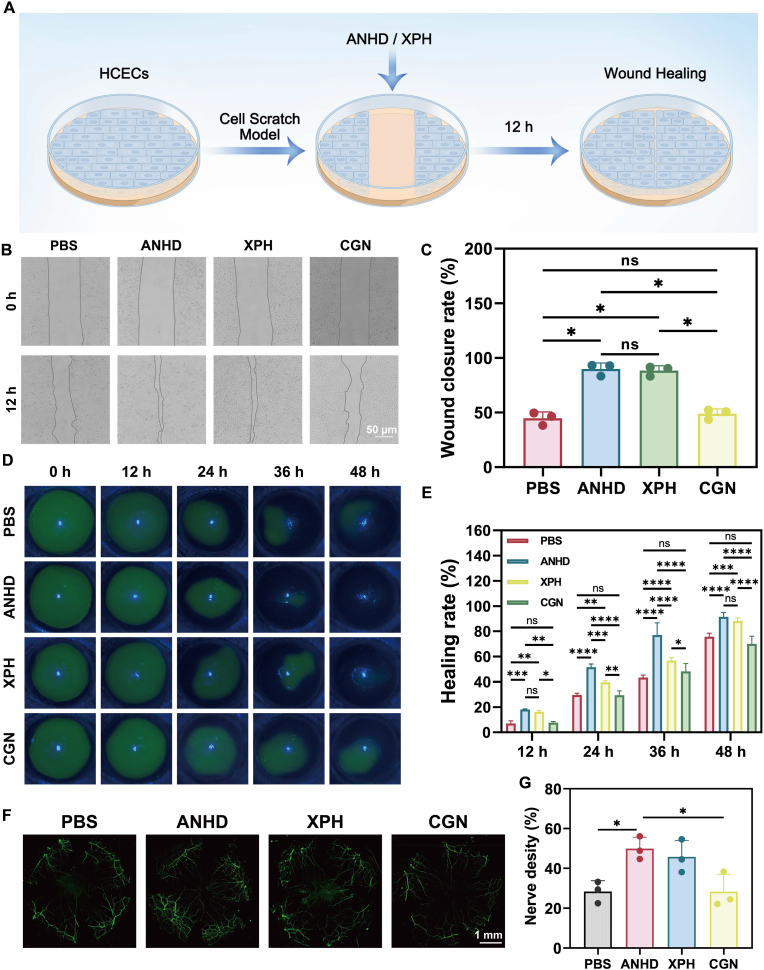
**The pro-healing performance of ANHD.** (A) Schematic of the scratch wound assay. (B) Representative images and (C) quantified migration rates of HCECs 12 h post-scratch after PBS, CGN, XPH, and ANHD treatment (scale bar, 50 μm). (D) Fluorescein-stained corneal wounds at predetermined time points post treatment by PBS, ANHD, CGN, and XPH and (E) statistical analysis of healing ratios. (F) Corneal nerve staining and (G) quantitative analysis of nerve density in epithelial injured mice on day 7 after different treatments (scale bar, 1 mm).

The effect of ANHD on corneal wound healing was initially investigated using cell migration assays (Fig. 7A). Scratch assays demonstrated that ANHD and XPH (0.1 mg/mL) significantly enhanced the cell migration within 12 h, while PBS and CGN exhibited negligible impact on corneal wound closure (Fig. 7B and C). We then assessed the *in vivo* pro-repair capacity of ANHD using corneal epithelial injury models. After 48 h post-operation, the ANHD and XPH groups had achieved complete wound healing, while the CGN group consistently showed the lowest healing rate at all time points. These results suggested XPH promoted corneal wound healing, while CGN might impede it (Fig. 7D and E). To further investigate the role of ANHD in promoting corneal nerve repair, neurofilament protein staining was performed on mouse corneas 7 days post-intervention (Fig. 7F). In the control and CGN groups, minimal nerve growth towards the central cornea was observed. In contrast, the ANHD and XPH groups exhibited significant nerve regeneration directed towards the central cornea, accompanied by a marked increase in nerve density (Fig. 7G). These results suggested that ANHD effectively promoted the repair of both corneal epithelial and neural damage.

### 3.7 *In vivo* BK treatment

**Fig. 8:**
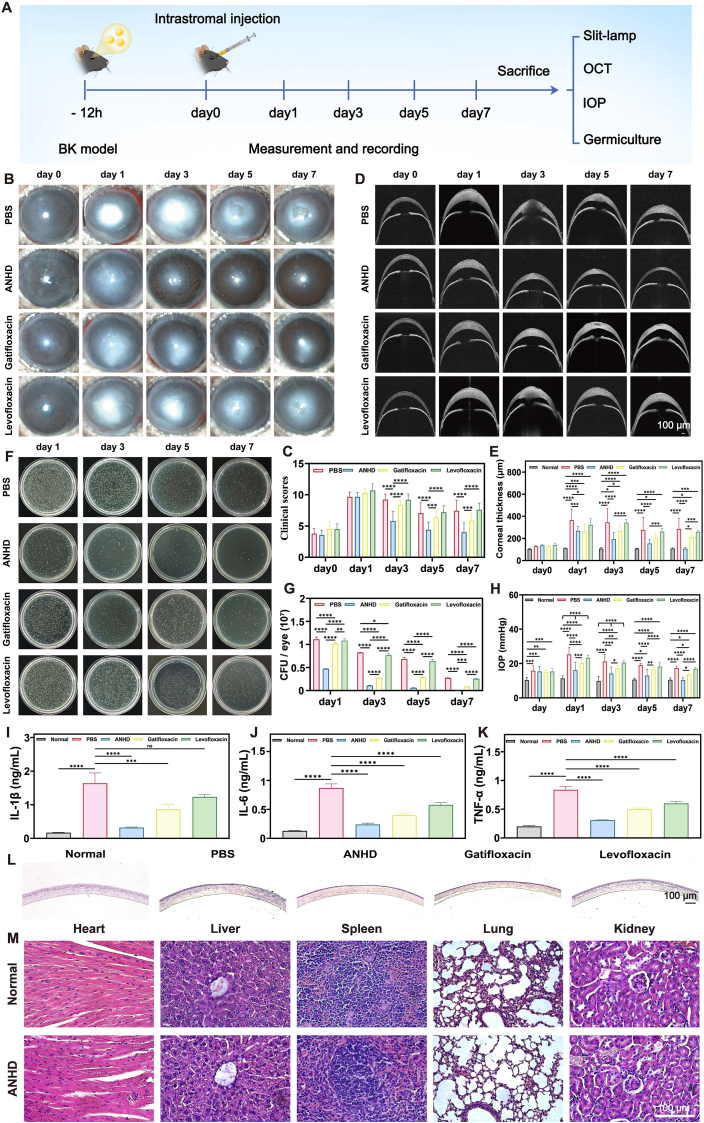
Therapeutic efficacy of ANHD in BK. (A) BK model establishment and treatment protocol. (B) Representative slit-lamp images of MDR *S. epidermidis*-infected corneas (*n* = 5) treated with PBS, ANHD, gatifloxacin, or levofloxacin on days 0, 1, 3, 5, 7. (C) Clinical severity scoring of corneal infection. (D) Central corneal thickness by AS-OCT, and (E) its quantitative analysis on days 0, 1, 3, 5, 7. (F) CFU counts of *S. epidermidis* in ocular surface secretions on days 1, 3, 5, 7 post-treatments, and (G) its quantitative analysis of bacterial survivals. (H) Quantitative IOP measurements after treatments on days 0, 1, 3, 5, and 7. (I–K) Expression levels of IL-1β, IL-6, and TNF-α in corneas after treatment by PBS, ANHD, gatifloxacin, or levofloxacin on day 7 post infection (*n* = 5). H&E-stained (L) corneal sections and (M) visceral histopathology of heart, liver, spleen, lung, and kidney (scale bar, 100 μm, *n* = 3) on day 7 post-infection after treatment by ANHD (*n* = 5).

The *in vivo* antibacterial efficacy of ANHD was evaluated using a murine BK model over 7 days (Fig. 8A). Slit-lamp imaging, ocular secretion cultures, central corneal thickness, and IOP were assessed and compared among the PBS, ANHD, gatifloxacin, and, levofloxacin groups. Similar clinical scores on day 0 across groups indicated comparable initial wound severity. Throughout the observation period, the PBS group consistently displayed severe corneal damage with marked opacity and swelling. In contrast, both ANHD and gatifloxacin treatments yielded significantly lower clinical scores compared to the PBS group (p < 0.01), with the superior efficacy of ANHD over gatifloxacin (Fig. 8B and C). In addition, the normal group maintained stable thickness throughout the period, while the control, levofloxacin, and gatifloxacin groups showed significantly higher levels than the normal group on days 1, 3, 5, and 7 (Fig. 8D and E). Fig. 8F and G shows bacterial culture results from ocular secretions, with CFU analysis confirming superior efficacy of ANHD. PBS, levofloxacin, and gatifloxacin groups showed significantly higher bacterial counts than ANHD on days 1, 3, 5, 7. ANHD-treated corneas showed increased thickness on day 1 but nearly normalized by day 7. Meanwhile, the control, levofloxacin, and gatifloxacin groups showed significantly higher IOP than both the normal and ANHD groups on day 7 (Fig. 8H).

In addition, ELISA analysis revealed obviously higher IL-1β, IL-6, and TNF-α levels in the control, levofloxacin, and gatifloxacin groups than those in the normal and ANHD groups (Fig. 8I–K). The H&E staining results showed that obvious corneal epithelial edema, thickening of interstitial layer, and infiltrated inflammatory cells in the PBS and levofloxacin groups (Fig. 8L). In the gatifloxacin group, the corneal epithelium showed mild corneal epithelial edema. The H&E staining of the ANHD group was similar to those of normal corneas, with no obvious inflammation or edema, indicating the therapeutic effect of ANHD in reducing inflammation and promoting repair. In addition, H&E staining of the heart, liver, spleen, lungs and kidneys in the normal and ANHD groups revealed the similar intact structures of the two groups with no significant differences (Fig. 8M). We further investigated the efficacy of ANHD in the treatment of *Pseudomonas aeruginosa* induced bacterial keratitis, and confirmed its therapeutic effect (Fig. S14, supporting information). These results demonstrated the superior antibacterial efficacy of ANHD, which combined antibacterial, ROS clearing, corneal reinforce, and tissue-repair functions in regionalized hydrogel cross-linked corneas.

**Fig. 9:**
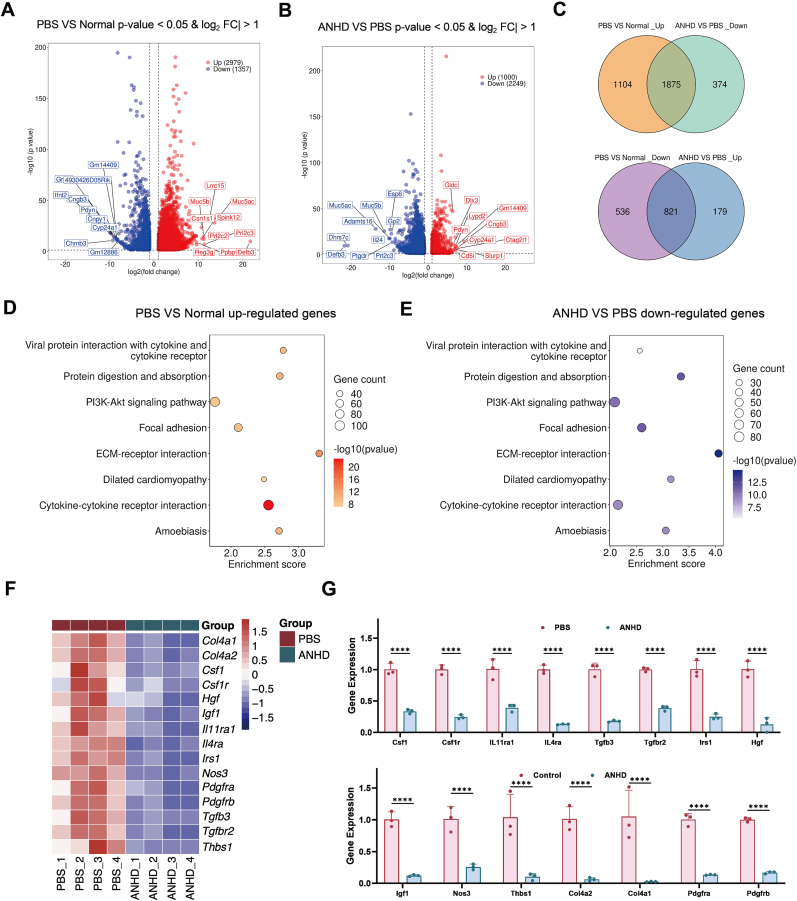
**Antibacterial mechanism of ANHD.** Volcano plots showing the DEGs from the corneal samples in (A) PBS *vs*. normal and (B) PBS *vs*. ANHD groups by RNA-seq analysis (Red, upregulated; blue, downregulated). (C) Venn diagram exhibiting overlap of DEGs between pathogenesis (PBS/normal) and treatment (ANHD/PBS) processes of BK. Bubble graph of the co-enriched pathways in both (D) pathogenesis and (E) treatment processes (dot size, gene count). (F) Heat map of 18 genes associated with cytokine-cytokine receptor interaction (*Csf1*, *Csf1r*, *Il11ra1*, *Il4ra*, *Tgfb3*, *Tgfbr2*), PI3K-Akt (*Hgf*, *Igf1*, *Irs1*, *Nos3*), ECM-receptor interaction (*Col4a1*, *Col4a2*, *Thbs1*, *Itgb1*), and focal adhesion (*Flna*, *Pdgfrb*, *Pdgfra*) pathways. (G) qRT-PCR validation of 18 representative genes in the PBS and ANHD groups.

To explore the potential antibacterial mechanism of ANHD, de novo RNA sequencing technology was used for identification of the differentially expressed genes (DEGs) after ANHD treatment. Principal component analysis (PCA) was performed by investigating the genes expression levels in the normal, PBS, and ANHD group (Fig. S15, supporting information). The results revealed that the specimens in these groups exhibited good independence. During the pathogenesis of BK, 4336 DEGs were identified by comparing the samples of the PBS group with the normal group, among which 31.3 % of the genes showed decreased expression (Fig. 9A). Meanwhile, during the treatment process, by comparing the samples of the ANHD group with the PBS group, 3249 DEGs were identified, of which 69.2 % of the genes showed decreased expression (Fig. 9B). Further analysis of the transcriptomic data indicated that a total of 2696 genes were involved in the both pathogenesis and treatment of BK, among which 30.4 % of the genes showed decreased expression during the pathogenesis and increased expression during the treatment (Fig. 9C). Similarly, eight pathways were enriched in the Kyoto Encyclopedia of Genes and Genomes (KEGG) analysis of the pathogenesis (Fig. 9D) and treatment (Fig. 9E) processes of BK. Among them, cytokine-cytokine receptor interaction, PI3K-Akt signaling pathway, ECM-receptor interaction, and focal adhesion were associated with anti-inflammatory, antioxidant, anti-scar, and tissue regeneration processes, respectively. Additionally, the clustering analysis of the main DEGs in four pathways was presented in the form of a heatmap, depicting the expression levels of different genes in the PBS and ANHD groups (Fig. 9F). The results revealed that these genes were down-regulated after ANHD treatment, and further confirmed by qRT-PCR analysis (Fig. 9G).

In our previous work, we introduced the nanozyme into infectious keratitis treatment by developing a composite hydrogel eye drop that exhibited good water dispersibility, peroxidase-like activity, and biocompatibility, and demonstrated efficacy against both *Pseudomonas aeruginosa* and MDR strains [55]. In recent years, MXenes and platinum-decorated V_2_C MXenes with NIR-II-enhanced nanozymic activity, iron-doped nanozymes with localized ROS generation capacity, and multimetallic nanozymes have been developed for anti-infective therapy [[56], [57], [58], [59]]. While these approaches have shown promising therapeutic outcomes, however, no consideration was given to eliminate the excessive oxidative stress that occurred during the infection process in these studies. To address this challenge, we developed a single-injection therapeutic strategy by engineering antibiotic nanozymes with SOD/CAT activities into a hydrogel matrix for *in situ* CXL. These nanozymes, synthesized from cerium nitrate and gatifloxacin, concurrently exerted antibacterial and ROS scavenging effects, thereby alleviating oxidative stress and inflammation. The ultra-small size of nanozymes and the injectability of hydrogel facilitated the uniform distribution of nanozymes throughout the corneal stroma. In addition, the combination of two precursors in nanozymes could reduce the antibiotic dosage, which may potentially lower the risk of antibiotic resistance. Besides, xanthan gum-based hydrogel could promote corneal wound healing, and reinforce corneal mechanical strength. Therefore, the ANHD represented a promising strategy for managing BK *via* a single administration.

## 4 Conclusions

In the study, an antibiotic nanozyme-incorporated polysaccharide hydrogel was developed for hybrid CXL therapy. The hydrogel composite exhibited synergistic multifunctional therapeutic effects, providing simultaneous antibacterial, antioxidant, pro-repair, and stroma-reinforcing activities within the corneal microenvironment, and showed potent efficacy against MDR *S. aureus* induced keratitis. Notably, the single-injection strategy, administered directly into the corneal stroma, eliminated the need for frequent medication. Furthermore, the hydrogel composite also demonstrated favorable biodegradation and biocompatibility. These results highlighted the potential of ANHD in the clinical treatment of bacterial keratitis. It is expected that diverse antimicrobial or immunomodulatory agents will be further integrated with functionalized nanomaterials to expand the treatment options for ocular infection diseases.

## CRediT authorship contribution statement

**Hongwei Wang:** Writing – original draft, Conceptualization. **Yuxin Liu:** Writing – original draft, Software, Methodology. **Li Ma:** Validation, Methodology. **Xiaoyan Sun:** Data curation. **Na Li:** Validation, Methodology. **Xin Sui:** Methodology. **Xia Qi:** Methodology. **Shengqian Dou:** Software, Formal analysis. **Tan Li:** Data curation. **Weiyun Shi:** Writing – review & editing, Supervision, Funding acquisition. **Ting Wang:** Writing – review & editing, Supervision, Funding acquisition.

## Declaration of competing interest

The authors declare that they have no known competing financial interests or personal relationships that could have appeared to influence the work reported in this paper.

## Data Availability

Data ·will be made available on request.
