# Supplementary material for: Antibiotic nanozyme hydrogel depot for single-injection MDR bacterial keratitis therapy via localized antibacterial, pro-healing and corneal reinforcement
Source: Mater Today Bio. 2025 Dec 3;36:102602. doi: 10.1016/j.mtbio.2025.102602 (PMC12757459; doi:10.1016/j.mtbio.2025.102602)
Supplement: Multimedia component 1 [file mmc1.docx]

Supporting information

Antibiotic nanozyme hydrogel depot for single-injection MDR bacterial keratitis therapy *via* localized antibacterial, pro-healing and corneal reinforcement

Hongwei Wang^a,c,d＃^, Yuxin Liu^a,b,d＃^, Li Ma^a,c,d^, Xiaoyan Sun^a,c^, Na Li^a,b,d^, Xin Sui^a,e^, Xia Qi^a,c,d^, Shengqian Dou^a,c,d^, Tan Li^a,c,d^, Weiyun Shi^a,b,d*^, Ting Wang^a,b,d*^

^a^State Key Laboratory Cultivation Base, Shandong Key Laboratory of Eye Diseases, Eye Institute of Shandong First Medical University, Qingdao, China

^b^Eye Hospital of Shandong First Medical University, Jinan, China

^c^Qingdao Eye Hospital of Shandong First Medical University, Qingdao, China

^d^School of Ophthalmology, Shandong First Medical University, Jinan, China

^e^The First School of Clinical Medicine, Binzhou Medical University, Binzhou, China

^＃^These authors contributed equally to the work

^*^ To whom correspondence should be addressed:

Prof. Weiyun Shi, E-mail: [weiyunshi@163.com](mailto:weiyunshi@163.com)

Prof. Ting Wang, E-mail: [wt-ting@163.com](mailto:wt-ting@163.com)

**Experimental Section**

**Swelling kinetics**

The swelling behavior of XPH was assessed at 6-hour and 12-hour time points. At each designated interval, XPH was immersed in phosphate-buffered saline (PBS, pH 7.4) and subsequently weighed. Prior to immersion, the initial dry weight of the XPH was recorded and then subtracted from the measured weight. The swelling ratio was calculated using the following formula: $\text{swelling}\text{ }\text{ratio}\text{ (\%)}\text{ }\text{=}\frac{\text{W}_{\text{final weight}}\text{ - }\text{W}_{\text{initial weight}}}{\text{ }\text{W}_{\text{initial weight}}}\text{×100}$

Where *W*_final_ _weight_ represents the measured weight of XPH after immersion into the PBS for a certain period, and *W*_initial_ _weight_ indicates the initial weight of XPH.

**Determination of acetylation degree**

Acetylation degree was determined using the following procedure. In brief, 20.0 mg of the prepared xanthan gum was added to 10.0 mL of 0.01 mol/L NaOH solution. Then, the mixture was heated to 50 °C and maintained for 2 hours. Methyl orange was used as the indicator and 0.01 mol/L HCl solution was used to titrate and neutralize the excess NaOH. H_2_O was used as the control group. The acetylation degree was calculated using the equation: $\text{acetyl}\text{ }\text{ (}\text{\%)}\text{ }\text{=}\frac{\left( \text{V}_{\text{blank}}\text{ - }\text{V}_{\text{sample}} \right)\text{ × }\text{N}_{\text{HCl }}\text{×}\text{ }\text{M}_{\text{acetyl}}}{\text{m}}\text{×100}$

where *V*_blank_ and *V*_sample_ represent the volumes of HCl consumed by the blank and sample, respectively, *M*_acetyl_ is 43 g/mol, *N*_HCl_ represents the normality of HCl.

**Quantification of aldehyde content**

The aldehyde content of OXG was evaluated using the acid-base titration method. Briefly, 0.1 g of xanthan gum and the freeze-dried OXG powder were dissolved in 20.0 mL of distilled water. 10.0 mL of hydroxylamine hydrochloride solution (0.25 mol/L) was added to each solution and stirred magnetically at 40 °C for 2 hours. Finally, the reaction mixture was titrated with 0.5 mol of NaOH solution. The aldehyde content was evaluated according to equation: $\text{alde}\text{hyde}\text{ }\text{content}\text{ }\left( \text{\%} \right)\text{=}\frac{\left( \text{V}_{\text{b}}\text{-}\text{V}_{\text{a}} \right)\text{×}\text{M}_{\text{NaOH}\text{×}\text{29}}}{\text{m}\text{×}\text{1}\text{000}}\text{×100}$

Where the volumes of NaOH solution for titrating XG and OXG are denoted as *V*_a_ and *V*_b,_ respectively. *M*_NaOH_ = 0.5 mol/L, and *m* is the dry weight of the OXG.

**Hemolytic activity assay**

The rabbit blood (10.0 mL) was freshly collected in an anticoagulant-treated tube, defibrinated by stirring with a glass rod for 10 minutes, diluted with saline and then centrifuged at 1500 ×g for 15 minutes. After centrifugation, the supernatant was carefully aspirated, and the red blood cells (RBC) pellet was washed three times with PBS to prepare a 4% RBC suspension. The hemolytic activity was evaluated using the following groups (*n*=3 per group): negative control, 200.0 µL 0.9% saline+200.0 RBCs; positive control, 200.0 μL 1% Triton X-100+200.0 RBCs; test groups, 200.0 ANHD extracts (1.0, 0.1, 0.01 mg/mL)+200.0 RBCs. All samples were incubated at 37 °C for 1 hour, followed by centrifugation (1000 ×g, 10 minutes). The absorbance of the supernatant was measured at 545 nm using a microplate reader. The hemolysis rate was calculated using the following formula: Hemolysis (%) = (OD_sample_ - OD_negative_)/(OD_positive_ - OD_negative)_×100%, where OD_positive_ and OD_negative_ represent 1% Triton X-100 and saline, respectively.

**Figures**

**Figure S1.** The gel permeation chromatography curve of xanthan gum (mobile phase, PBS; 40 °C, flow rate, 1.0 mL/min).


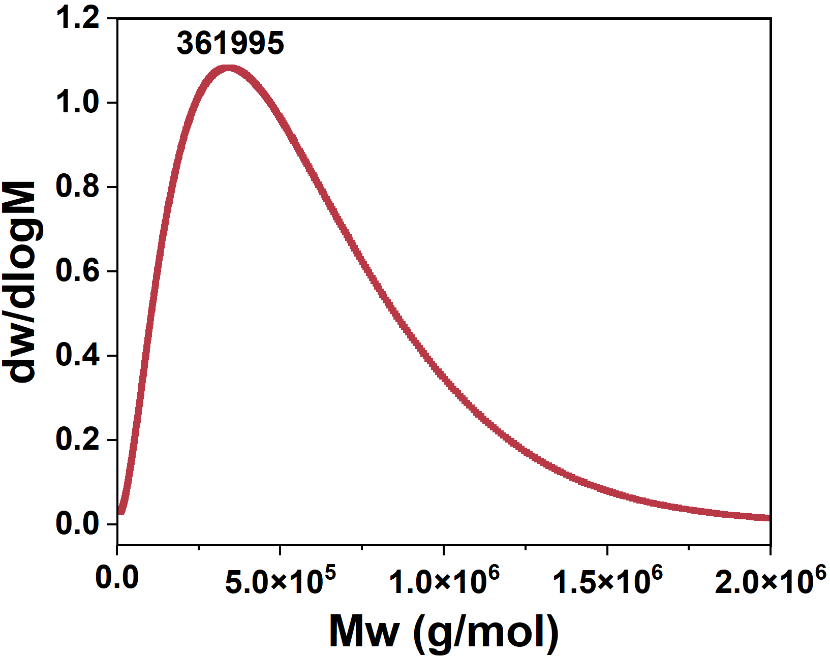


**Figure S2.** The optical photograph of XPH.


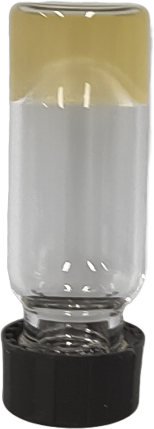


**Figure S3.** Standard curve of gatifloxacin plotted by UPLC.


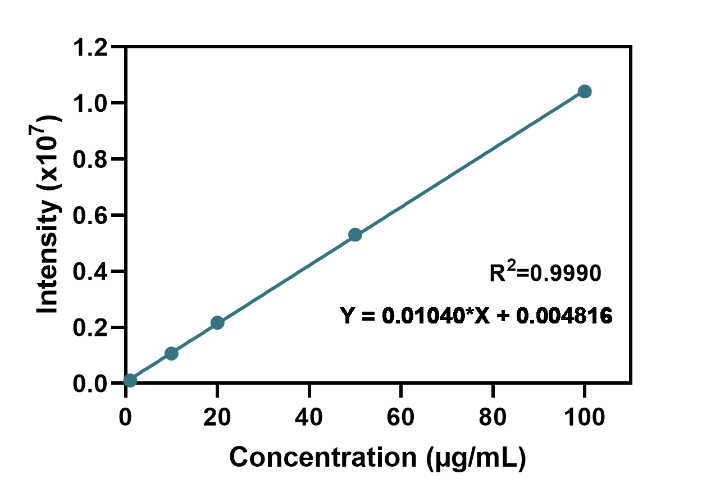


**Figure S4.** The swelling ratios of XPH at 6 hours and 12 hours, respectively.

**
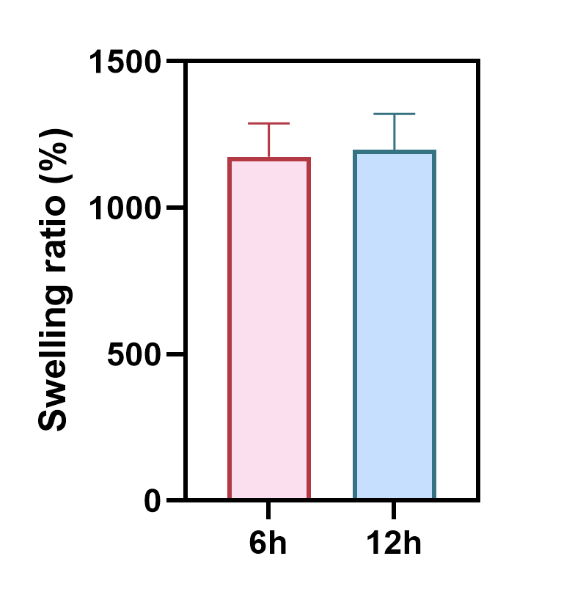
**

**Figure S5.** Viscosity-shear rate curves of XPH and ANHD.


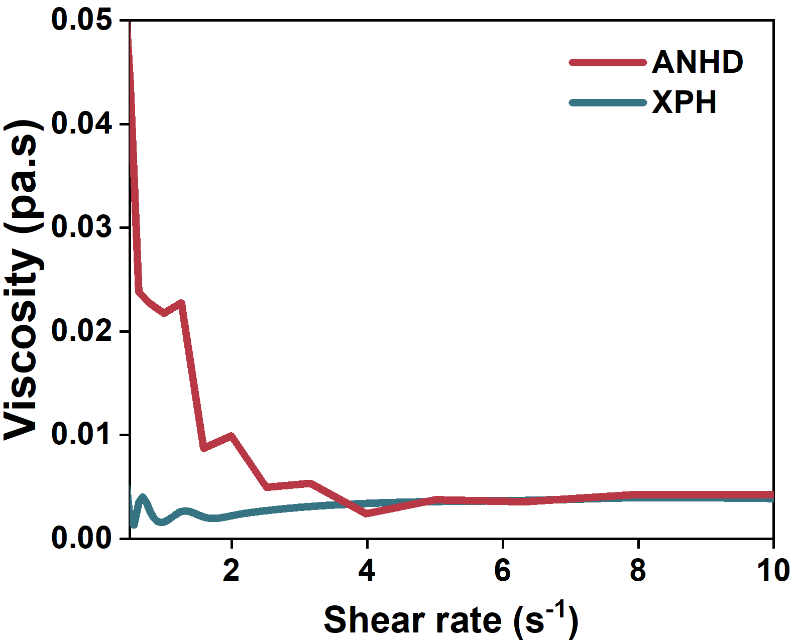


**Figure S6.** Biocompatibility assessment. (A) Central corneal thickness and (B) intraocular pressure of mice after treatment by CGN, XPH, and ANHD.


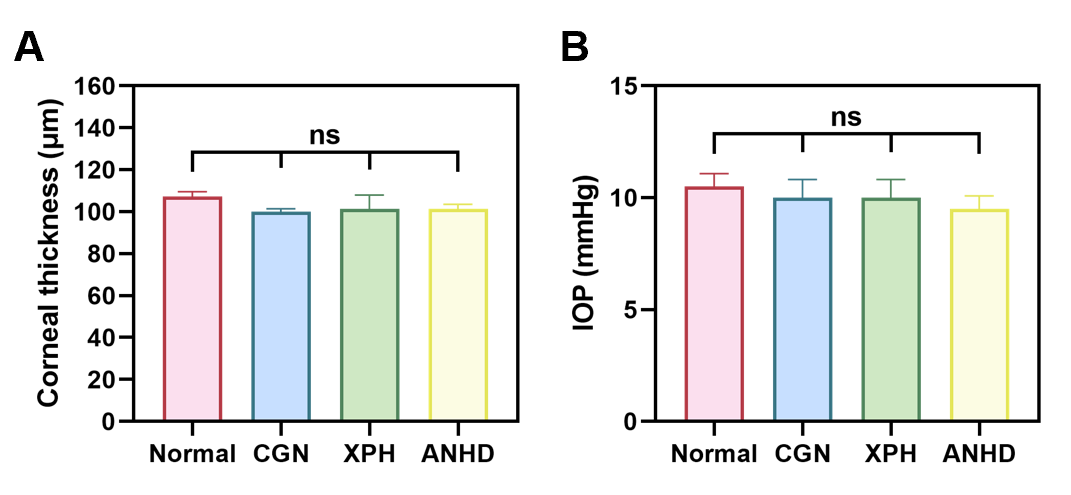


**Figure S7.** (A, B) The hemolysis test of ANHD. (C) Detection of white blood cells, red blood cells and platelets.


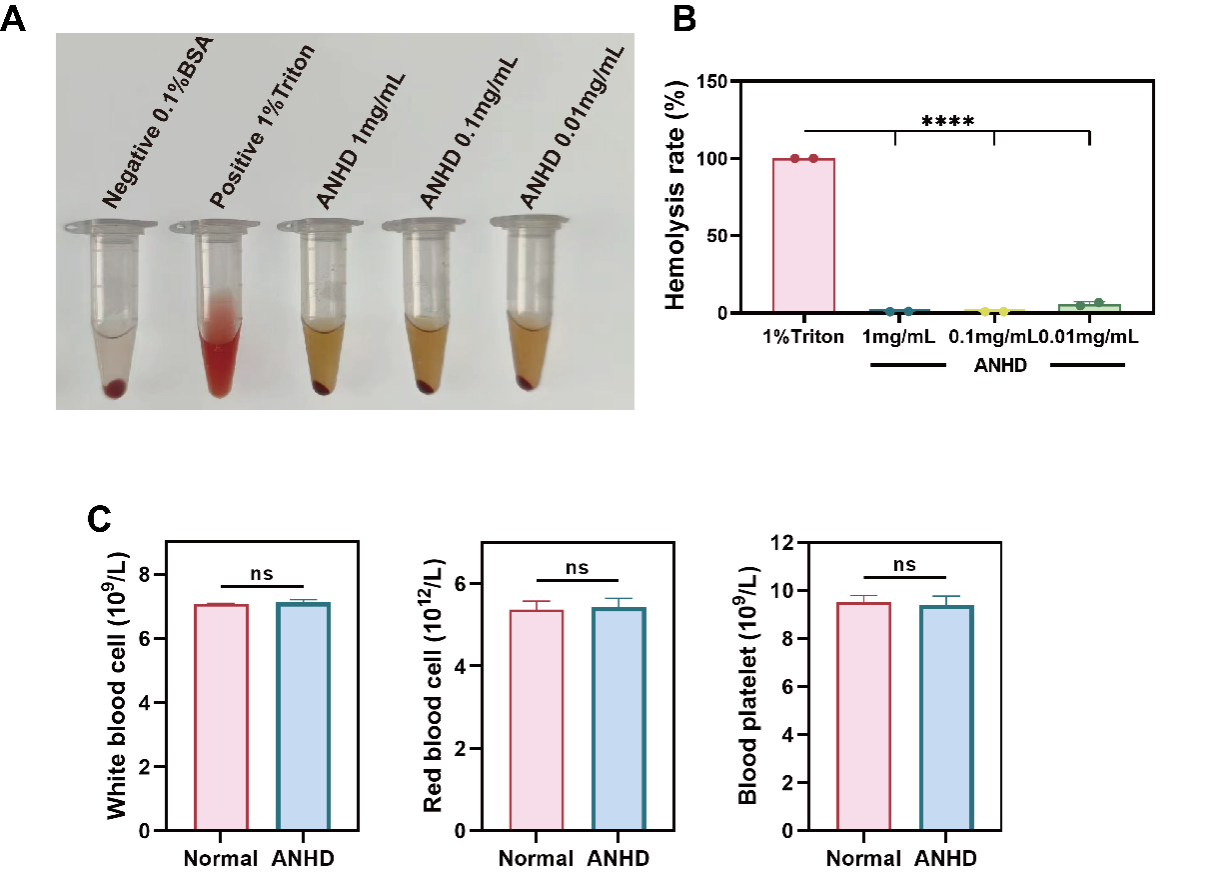


**Figure S8.** The capacity of OXG for scavenging (A) H_2_O_2_, (B) O_2_^•−^, and (C) ·OH.

**
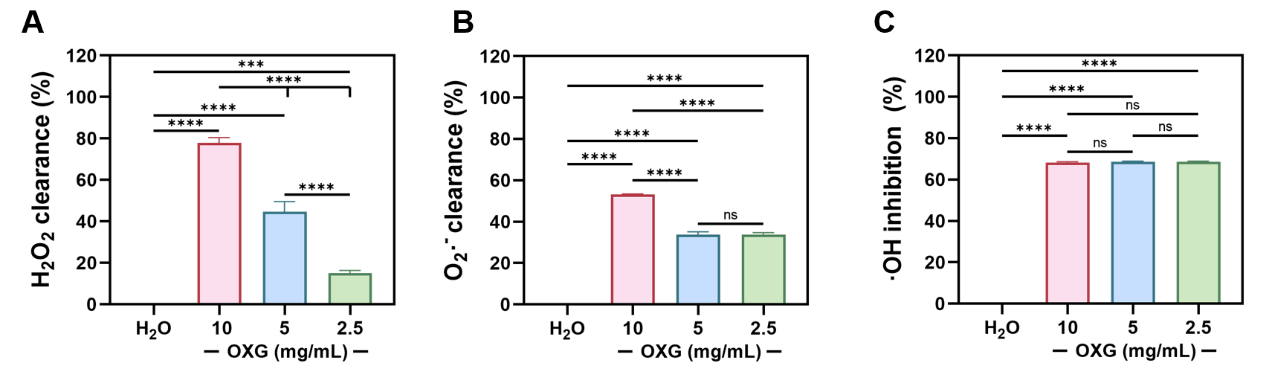
**

**Figure S9.** The H_2_O_2_ clearance rates of CGN at pH values of 5, 6, and 7.


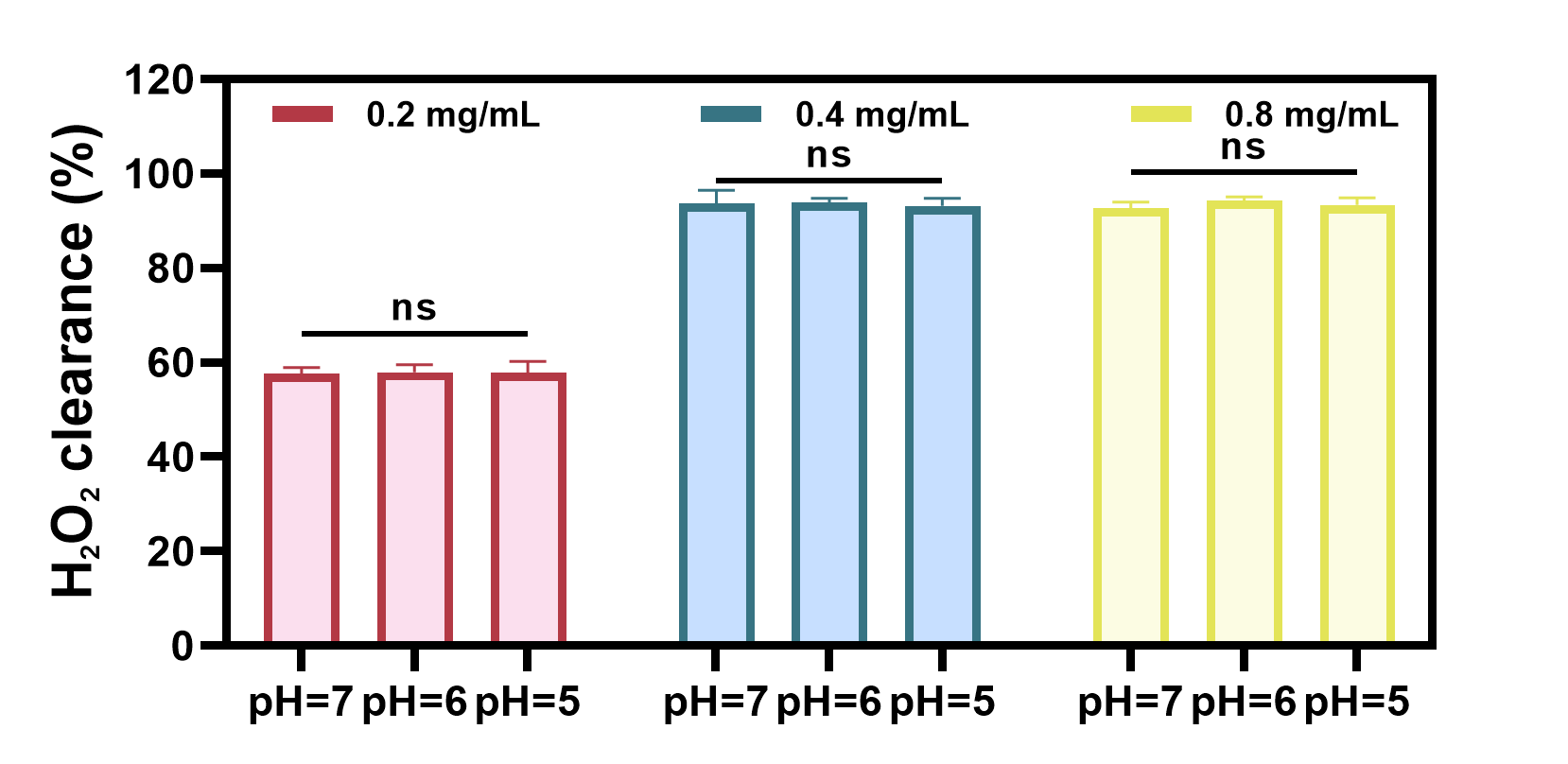


**Figure S10.** Bright field images of RAW 264.7 after treatments by LPS and LPS+ANHD.


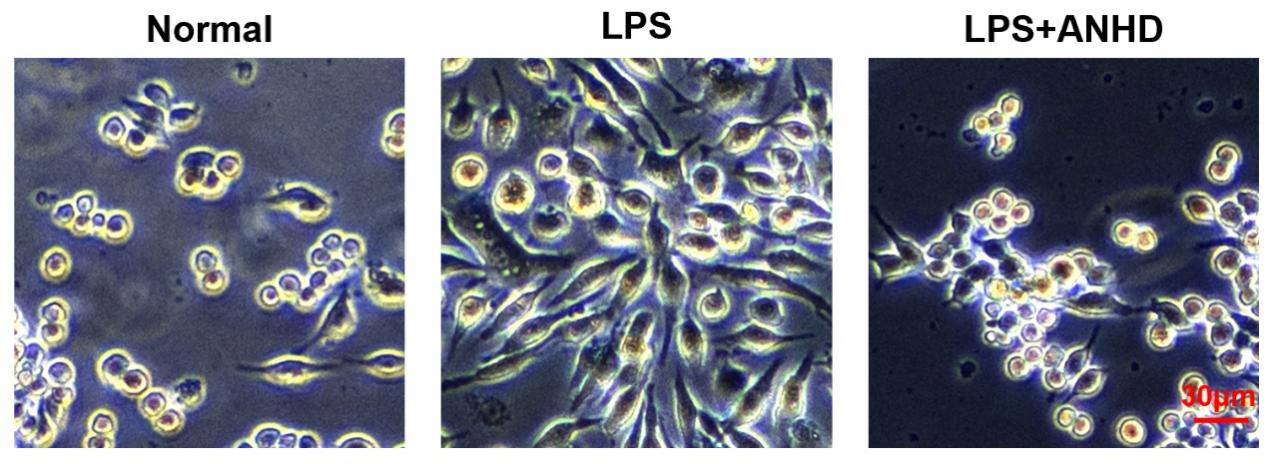


**Figure S11.** Expression levels of IL-1β, IL-6 and TNF-α in the corneas of the PBS and ANHD groups by the qRT-PCR analysis.


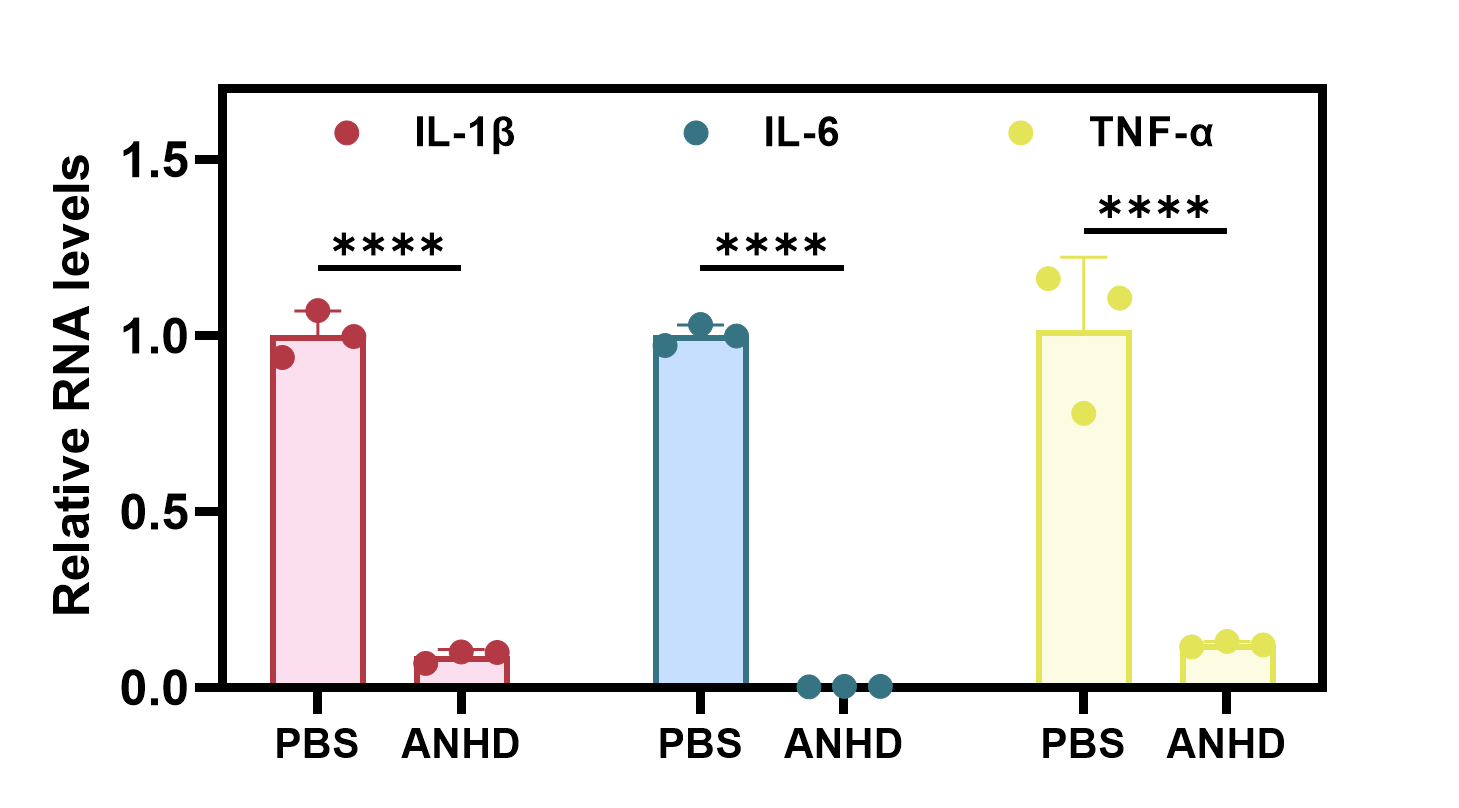


**Figure S12.** Quantitative analysis of the biofilms *via* CV staining in the four groups.

**
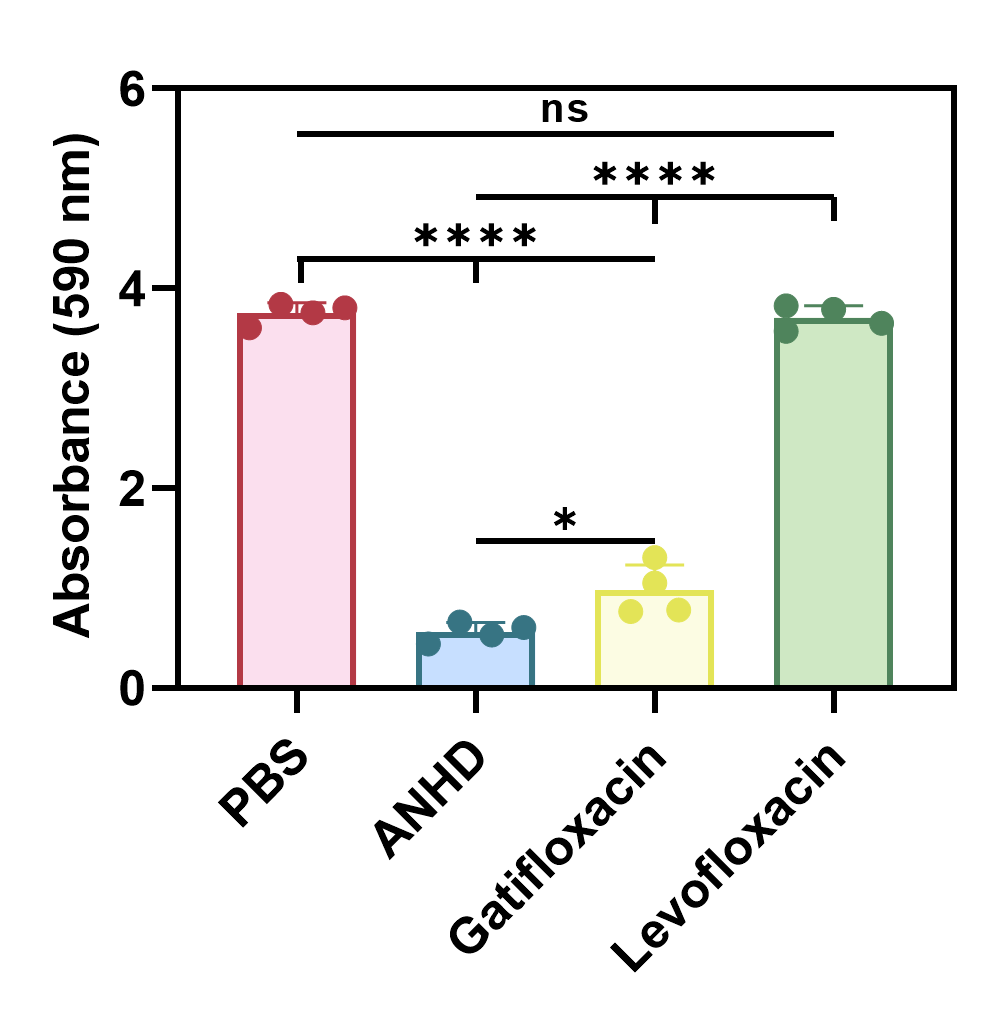
**

**Figure S13.** Standard curves of gatifloxacin plotted by LC-MS/MS.


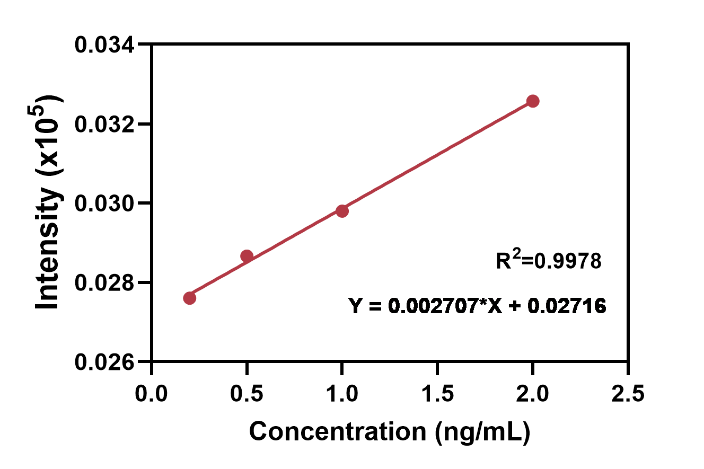


**Figure S14.** Slit-lamp examination (A) and clinical scoring (B) of *Pseudomonas aeruginosa* infected corneas.


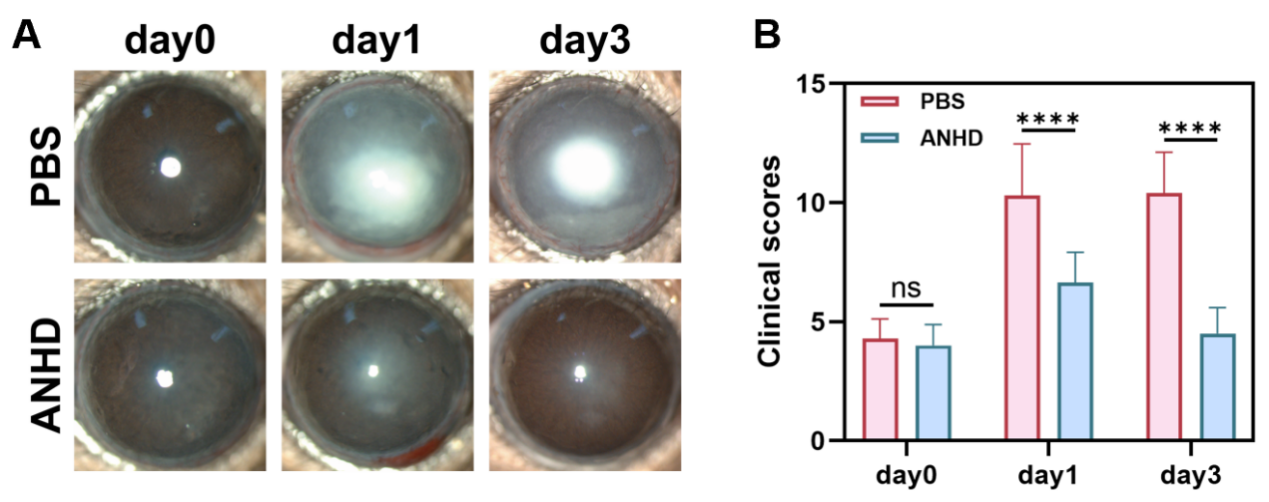


**Figure S15.** Principal component analysis (PCA) of the gene expression levels in the normal, PBS, and ANHD groups.


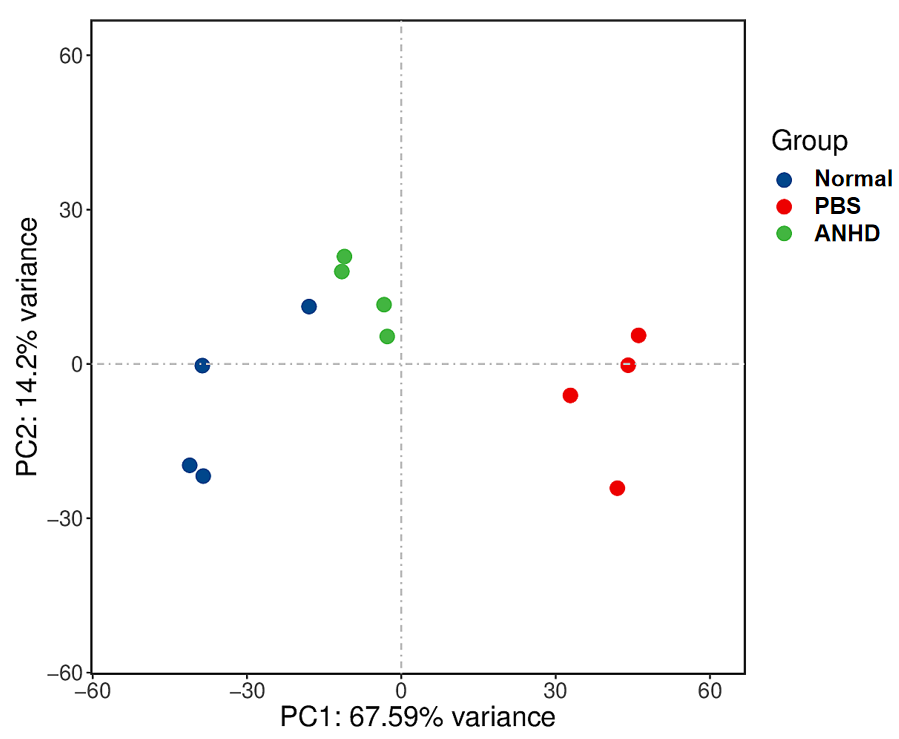


**Table S1.** The MS parameters for quantitative analysis of gatifloxacin.

| Compound | Formula | Parent (*m*/*z*) | Cone voltage (V) | Daughters  (*m*/*z*) | Collision energy (V) | Ion mode |
| --- | --- | --- | --- | --- | --- | --- |
| Gatifloxacin | C_19_H_22_FN_3_O_4_ | 393.13 | 2 | 129.09 | 34 | ES^+^ |

**Table S2.** Clinical scoring of infectious keratitis.

| Score | Area of corneal opacity |  | Density of corneal opacity |  | Surface regularity |
| --- | --- | --- | --- | --- | --- |
| 1  2 | 1%-25%  26%-50% |  | Slight cloudiness, outline of iris and pupil discernable  Cloudy,but the outlines of the iris and pupil remain visible |  | Slight surface irregularity  Rough surface, some swelling |
| 3 | 51%-75% |  | Cloudy, opacity not uniform |  | Significant swelling, crater or serious descemetocele formation |
| 4 | 76%-100% |  | Uniform opacity |  | Perforation or descemetocele |
